# Climate change impacts to foraging seascapes for a highly migratory top predator

**DOI:** 10.1186/s40462-025-00558-1

**Published:** 2025-05-09

**Authors:** Barbara Muhling, Stephanie Snyder, Elliott L. Hazen, Rebecca Whitlock, Jong-Yeon Park, Charles A. Stock, Barbara A. Block

**Affiliations:** 1 Institute of Marine Sciences Fisheries Collaborative Program, University of California, Santa Cruz, Santa Cruz, CA USA; 2 Southwest Fisheries Science Center, National Oceanic and Atmospheric Administration, La Jolla, CA USA; 3 Thomas More University, Crestview Hills, KY USA; 4 Southwest Fisheries Science Center, National Oceanic and Atmospheric Administration, Monterey, CA USA; 5 Department of Aquatic Resources, Swedish University of Agricultural Sciences, Stångholmsvägen 2, 178 93 Drottningholm, Sweden; 6 Department of Earth and Environmental Sciences, Jeonbuk National University, Jeonju, Jeollabuk-Do Republic of Korea; 7 Geophysical Fluid Dynamics Laboratory, National Oceanic and Atmospheric Administration, Princeton, NJ 08540 USA; 8 Hopkins Marine Station, Stanford University, Pacific Grove, CA 93950 USA

**Keywords:** North Pacific albacore, Foraging ecology, Tuna migration, Highly migratory species, Climate change

## Abstract

**Background:**

Climate change is impacting the distribution and movement of mobile marine organisms globally. Statistical species distribution models are commonly used to explain past patterns and anticipate future shifts. However, purely correlative models can fail under novel environmental conditions, or omit key mechanistic processes driving species habitat use.

**Methods:**

Here, we used a unique combination of laboratory measurements, field observations, and environmental predictors to investigate spatial variability in energetic seascapes for juvenile North Pacific albacore tuna (*Thunnus alalunga*). This species undertakes some of the longest migrations of any finfish, but their susceptibility to climate-driven habitat changes is poorly understood. We first built a framework based on Generalized Additive Models to understand mechanisms of energy gain and loss in albacore, and how these are linked to ocean conditions. We then applied the framework to projections from an ensemble of earth system models to quantify changes in thermal and foraging habitats between historical (1971–2000) and future (2071–2100) time periods.

**Results:**

We show how albacore move seasonally between feeding grounds in the California Current System and the offshore North Pacific, foraging most successfully in spring and summer. The thermal corridors used for migration largely coincide with minimum metabolic costs of movement. Future warming may result in loss of favorable thermal habitat in the sub-tropics and a reduction in total habitat area, but allow increased access to productive and energetically favorable sub-arctic ecosystems. Importantly, while thermal considerations suggest a loss in habitat area, forage considerations suggest that these losses may be offset by more energetically favorable conditions in the habitat that remains*.* In addition, the energetic favorability of coastal foraging areas may increase in future, with decreasing suitability of offshore foraging grounds. Our results clearly show the importance of moving beyond temperature when considering climate change impacts on marine species and their movement ecology.

**Conclusions:**

Considering energetic seascapes adds essential mechanistic underpinning to projections of habitat gain and loss, particularly for highly migratory animals. Overall, improved understanding of mechanisms driving migration behavior, physiological constraints, and behavioral plasticity is required to better anticipate how climate change will impact pelagic marine ecosystems.

**Supplementary Information:**

The online version contains supplementary material available at 10.1186/s40462-025-00558-1.

## Introduction

Climate change is driving distribution shifts in mobile marine species worldwide [36, 62]. The impact of warming on species distribution shifts is well documented, as animals move poleward or into deeper waters to avoid physiological impacts of rising temperatures [19]. However, species may also move to follow aggregations of important prey [41, 76]. These shifts are less well understood but can interact with changes in thermal habitat suitability in complex ways. Different rates of species movements can lead to new community assemblages and altered trophic interactions, with flow-on effects for marine foodwebs [53, 69]. As ecosystems are reorganized, populations of animals targeted by fisheries can change in abundance and spatial availability. These changes can impact fishing opportunities and the portfolio of species available to fishing fleets, with potential socioeconomic repercussions for fishing-dependent communities [67].

Species distribution shifts thus have consequences for ecosystem structure, as well as natural resource management and human uses of the ocean. Anticipating future shifts can help fisheries and ecosystem-based management frameworks to be better prepared for ongoing climate change, and promote development of “climate-ready” governance [44]. Mathematical models that can predict species distributions from environmental conditions are key tools in this process, and have become increasingly popular in the past several decades (e.g., [45]). Distribution models can incorporate a range of predictors including physical variables (e.g., temperature, salinity: e.g., [50]) and biogeochemical variables (chlorophyll, dissolved oxygen: e.g., [21]). Occasionally these models include some representation of prey fields (e.g., [70]), but incorporation of predator–prey relationships is often limited by a lack observations on diet composition and prey distributions. Regardless of input predictors, species distribution models typically assume that animals have a set range of environmental conditions that they can tolerate based on their physiology. As a result, if spatial availability of this habitat shifts in response to climate forcing, mobile animals will move to follow it. This conceptual model can break down, however, if species move for reasons not well captured by simple statistical models. Such drivers can include reproductive phenology, following specific prey, or avoidance of predation [4, 9]. The spatial distribution of species that migrate long distances as part of their life cycles can be especially difficult to capture in simple statistical models, as these animals may have complex movement behaviors driven by cues unrelated to contemporaneous conditions.

Marine species have evolved a diverse array of migration behaviors to maximize reproductive success, maintain optimal energetics, enhance growth, and avoid predators, among other motivations [4]. Foraging migrations can allow predators to exploit spatiotemporally variable prey resources, tracking areas of high potential energy gain in time and space [2]. Migration timing often evolves to place animals in optimal habitat at their anticipated destination, based on historical patterns in seasonal prey concentrations, for example [9]. Long-distance migrations may involve periodic switches between relative resident behaviors within favorable habitats, and rapid, energetically costly movements between favorable habitats [57]. As a result of these complex drivers, highly migratory animals are not evenly or randomly distributed across their geographic ranges. Building models to predict the distributions of migratory animals, both historically and into the future, is therefore particularly challenging [45].

Understanding the mechanisms driving potential climate impacts on highly migratory species is a pressing problem [77]. As climate change leads to shifts in the availability of suitable thermal habitat and foraging resources for marine species, historically beneficial migration strategies may become maladaptive [65]. Seasonal seascapes of potential energetic costs and gains are likely to change, and spatiotemporal mismatches between predators and prey may result. Animals may need to shift the timing or paths of their migrations to effectively adapt, and maintain the energetic benefits of historical migratory behaviors [49]. A first step to anticipating these changes is to model future shifts in locations of optimal energy gain for migratory species, and show how these intersect with changes in thermal habitat.

Albacore tuna (*Thunnus alalunga*, albacore hereafter) is a highly migratory species distributed globally. They support substantial commercial and recreational fisheries throughout much of their geographic range [32, 54]. In the North Pacific Ocean, immature albacore (~ 2–5 years old) can migrate thousands of kilometers seasonally between coastal ecosystems in the California Current and Kuroshio Current, and the offshore North Pacific ([24, 54]: Supplementary Fig. 1). Migration behaviors can be highly variable among individual albacore, but the drivers of these movements are not well understood [57]. Juvenile albacore can forage across both epipelagic and mesopelagic environments [7, 57]. They are highly flexible foragers, targeting a variety of prey from small pelagic fishes (e.g. anchovy: *Engraulis mordax*) to mesopelagic cephalopods and small crustaceans such as krill [58]. Albacore are thus an ideal model species for considering how highly migratory marine animals may respond to climate-driven shifts in foraging environments and energetic seascapes. In this study, we investigate how foraging seascapes and thermal habitat may interact to determine the future distribution of juvenile albacore in the North Pacific.

We first describe a hybrid correlative-mechanistic modeling framework to predict distributions of thermal and energetic habitats for juvenile albacore in the North Pacific. A combination of laboratory measurements, field data, and environmental predictors are leveraged to develop a data-driven modeling framework linking ocean conditions to energetic seascapes. These models are then applied to projections from an ensemble of earth system models to quantify how habitat for albacore may change into the future. We compare habitat shifts based only on temperature to those considering foraging energetics, and highlight the most important contributors to uncertainty and error propagation within the modeling framework. We hypothesize that projected changes to albacore habitat defined using energetic seascapes will be spatially distinct and more heterogeneous from habitat defined using only temperature.

## Methods

Our study region covered the sub-tropical to sub-arctic latitudes of the North Pacific, where juvenile albacore are known to occur (Supplementary Fig. 1). The modeling framework used a combination of field observations from archival-tagged albacore, laboratory measurements of foraging and energy gain, and a suite of environmental predictors to define energetic seascapes. Each piece of the framework is described in more detail below.

### Predictive models (GAMs)

**Fig. 1:**
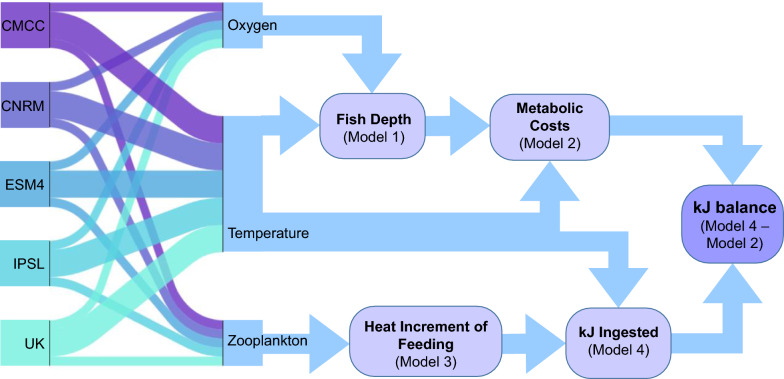
Modeling framework showing five earth system models providing three environmental predictors to inform the four Generalized Additive Models shown in Table 1. The 5 earth system models are CMCC-ESM2 [52], CNRM-ESM2-1 [71], GFDL-ESM4 [28], IPSL-CM6A-LR [15] and UKESM1-0-LL [72]: more details are given in the Supplementary Materials

Our framework combined a series of Generalized Additive Models (GAMs) to predict energetic costs and gains for juvenile albacore across the North Pacific Ocean (Table 1, Fig. 1). GAMs were selected due to their simplicity, flexibility, and ability to constrain partial responses to ensure biological realism. Models were constructed in the *mcgv* package [81] in R 4.4.0 [64], with relative predictor importance calculated in the *gam.hp* package [46]. GAMs were parameterized using either field-collected data (tagged fish) or laboratory data (captive fish), and then applied to outputs from earth system models.

**Table 1:** Summary of generalized additive models (GAMs) used to predict albacore depth, metabolic movement costs, Heat Increment of Feeding, and energy ingested. Predictors and % model deviance explained (%) are shown. Partial response plots are in the Supplementary Materials as indicated

Model no. (Fig. 1)	Model output	Biological data source	Predictors	% Dev. Expl	Partial response figure
1a	Fish depth (daytime, m)	Tagged wild albacore (1)	Upper 200 m temperature (°C) Moon phase (% illuminated) Depth of 3.5 ml L^−1^ dissolved oxygen	48.1	Supp. Figure 2
1b	Fish depth (nighttime, m)	Tagged wild albacore (1)	Upper 200 m temperature (°C) Moon phase (% illuminated) Depth of 3.5 ml L^−1^ dissolved oxygen	35.3	Supp. Figure 2
2	Metabolic movement costs (mg O_2_ kg^−1^ h^−1^)	Captive Pacific bluefin tuna (2)	Water temperature at depth of fish (°C) Fish swimming speed (body lengths s^−1^)	87.5	Supp. Figure 3
3	Heat Increment of Feeding (°C hour)	Tagged wild albacore (1)	Upper 200 m mesozooplankton (mg C m^−2^) Estimated fish length (cm) Day length (hours)	34.0	Supp. Figure 4
4	Energy Ingested (kJ)	Captive Pacific bluefin tuna (3)	Heat Increment of Feeding (°C hour) Water temperature at depth of fish (°C) Proportion of sardine in diet (%)	84.5	Supp. Figure 5

1: Muhling et al. [57]

2: Blank et al. [11, 12], Clark et al. [26]

3: Whitlock et al. [79]

GAMs 1 and 2 combine to predict the metabolic costs associated with each set of environmental conditions, while GAMs 3 and 4 combine to estimate the energy gained through foraging (Fig. 1). The difference yields a measure of net energetic favorability in kJ.

### Estimates of energetic costs

Archival tag records from 28 juvenile albacore were available through the Albacore Archival Tagging Program [24] and the TOPP (Tagging of Pacific Predators) project [13] (see Supplemental Methods). We first used these data to predict diel depth distributions of albacore, which inform the modeled ambient temperatures that they experience. Previous work has shown that albacore adjust their vertical distribution depending on environmental conditions and moon phase [33]. However, their response to these variables is strongly distinct between night and daytime, as deeper dives associated with foraging are concentrated during daylight hours [57]. *Models 1a and 1b* thus predicted the depth of tagged albacore during the day and night, using upper 200 m mean water temperature, the depth at which dissolved oxygen is 3.5 ml L^−1^, and moon phase (Table 1, Fig. 1). 3.5 ml L^−1^ is approximately the level below which epipelagic tunas such as albacore may experience physiological stress [17]. Temperature and oxygen were obtained from a data-assimilative retrospective physical ocean simulation integrated with a non-assimilative biogeochemical/plankton food web model [60], while moon phase was obtained from the *lunar* package in R [47].

Potential metabolic costs were calculated using ambient temperatures at the predicted depths of albacore, for daytime and nighttime separately. *Model 2* (Table 1, Fig. 1) predicts metabolic costs of movement (mg O_2_ kg^−1^ h^−1^) from a combination of water temperature and swimming speed (body lengths s^−1^), using laboratory observations from the closely-related juvenile Pacific bluefin tuna (*T. orientalis*, [11, 12, 26, 56]). Albacore swimming speeds were fixed at 1 body length s^−1^ during the nighttime and 2 body lengths sec^−1^ during daytime in the model, based on observations of captive tunas and diel behavior of tagged albacore [11, 57]. We converted metabolic costs (mg O_2_ kg^−1^ h^−1^) to kJ assuming that 1 mg O_2_ = 13.59 J [31, 42].

### Estimates of energy gains

To estimate daily energy intake in tagged albacore, we first estimated the Heat Increment of Feeding (HIF: *model 3*). HIF is the increase in visceral temperature due to specific dynamic action, or the heat output from the metabolic processes required to digest and assimilate a meal [20]. HIF has been quantified using observations of internal body temperature from surgically implanted archival tags in wild and captive bluefin tuna, and wild albacore [6, 57, 79, 80]. We calculated HIF as the area under the curve between the estimated baseline (“fasting”) body temperature and observed body temperature from the archival tags at 1-min intervals, across each 24-h period for each fish (for details see Muhling et al. [57] and Supplementary Methods). Juvenile albacore have diverse and flexible diets including finfish (e.g., anchovy, sardine (*Sardinops sagax*), saury *Cololabis saira*), cephalopods, and crustaceans, and their diet composition varies strongly in space and time [58]. However, Muhling et al. [57] showed that daily HIF in archival tagged wild albacore is positively correlated with model-based estimates of upper 200 m mesozooplankton biomass, as a proxy for overall prey availability. As juvenile albacore primarily forage during the day, seasonal changes in hours of daylight at each fish’s location may also influence the energy they can ingest. Growth in other tunas has also been observed to be highly seasonal (e.g., [22]). Lastly, field observations of tagged albacore and bluefin tuna show that HIF is greater in larger fish [57, 80]. *Model 3* (Table 1, Fig. 1) therefore predicts observed HIF in tagged wild albacore using upper 200 m mesozooplankton biomass (mg C m^−2^: from [60], estimated fish length (cm fork length), and day length (hours). Daily fish lengths were estimated assuming linear growth between the recorded lengths at release and recapture, based on the growth curve in Xu et al. [84].

Data from captive fish show that HIF is strongly correlated with the caloric value of a meal, but also modulated by diet composition and ambient temperature [79] (Supplementary Fig. 5). *Model 4* (Table 1, Fig. 1) therefore predicts kJ ingested from a combination of HIF, water temperature, and estimated proportion of finfish prey (e.g., sardine, saury, *Cololabis saira*) versus mollusk or crustacean prey (e.g. *Doryteuthis opalescens, Onychoteuthis borealijaponica*). As we cannot observe the diet composition of tagged wild fish, we used a fixed value of 70% finfish based on previous diet studies from both the nearshore and offshore eastern North Pacific [40, 58]. Losses from specific dynamic action (9.2%) and excretion/egestion (27%) were based on Estess et al. [31]. The daily kJ balance was thus the estimated kJ ingested minus losses from movement costs, specific dynamic action, and excretion/egestion. Times and places associated with higher/positive kJ balances are assumed to be energetically favorable, and where excess energy may be available for processes such as growth or maturation. While we did not directly estimate metabolic costs of somatic maintenance, growth or maturation (e.g., [22, 43]), we assumed that any energetic excess available each day would be available for these processes.

### Estimating daily energetic balance in tagged fish

We examined daily spatial variability in energetic gains, losses, and balances in all tagged fish using observations of ambient temperatures and HIF applied to *models 2 and 4* (Table 1, Fig. 1). To estimate metabolic costs of movement in tagged fish, we calculated horizontal distance traveled as the great-circle distance between consecutive daily positions, smoothed using a 7-day moving mean to reduce the effects of geolocation error [16]. We calculated vertical distance as half the total distance traveled in the vertical plane, assuming that descending movements require minimal energy [6]. The total daily distance traveled in three dimensions was then converted to BL s^−1^, using the estimated daily length of each fish. Ambient temperature was the mean daily external temperature recorded by the tag for each fish (across all depths). We used the length–weight relationship from Chen et al. [23] to convert estimated daily lengths of tagged fish to weights, and then converted metabolic costs (mg O_2_ kg^−1^ h^−1^) to kJ using relationships from Estess et al. [31]. HIF was converted into estimated kJ ingested using *model 4*.

### Projecting future changes in thermal and energetic habitats

Historical and future projections of environmental predictors were obtained from a suite of earth system models. We selected five models from the sixth phase of the Coupled Model Intercomparison Project (CMIP6) with reasonable skill in replicating spatial fields of mesozooplankton biomass [61]: CMCC-ESM2, CNRM-ESM2-1, GFDL-ESM4, IPSL-CM6A-LR, and UKESM1-0-LL (Supplementary Table 1 and Supplementary Methods). We extracted temperature (SST and upper 200 m mean temperature), depth at which dissolved oxygen is 3.5 ml L^−1^, and total upper 200 m mesozooplankton biomass from each earth system model and applied these to the framework shown in Fig. 1. We then quantified changes in metabolic costs, energetic gains, and overall daily energy balances between the historical (1971–2000) and future (2071–2100) periods under a moderate emissions scenario (SSP2-4.5), for each earth system model. We calculated values based on an albacore of 80 cm fork length and 11 kg weight, based on mean sizes of tagged albacore and sizes of fish used in laboratory experiments. An example of the modeling workflow applied to mean environmental fields for the historical period (1971–2000) is shown in Supplementary Fig. 6.

### Comparing thermal versus energetic habitat shifts

Juvenile albacore habitat is strongly delineated by SST [33, 74]. Many climate change impact studies use models based on temperature (with or without other predictors) to develop scenarios of species distribution shifts, and habitat gain or loss (e.g., [37, 50]). However, ecological outcomes of species distribution shifts depend not just on thermal habitat, but also on metabolic costs, and changes in foraging resources. To compare a temperature-only approach to the more complex energy seascapes approach developed here, we first quantified the seasonal gain and loss of albacore thermal habitat between the historical and future periods using only SST. We used a simple thermal envelope approach based on SSTs from the NOAA 0.25° Daily Optimum Interpolation Sea Surface Temperature (OISST) product, version 2.1 [39] associated with locations of tagged fish (Supplementary Figs. 1 and 7). This range (11–22 °C) is consistent with previous studies of albacore distribution from both tags and fishery-dependent data [33, 57]. SST-based habitat gain/loss was defined simply as the change in area (km^2^) of thermally favorable albacore habitat in the North Pacific Ocean between the historical and future periods, within each season. We then calculated the change in total daily kJ balance resulting from thermal habitat shifts. We calculated this metric using area-weighting (i.e., we multiplied the kJ balance for all grid cells within favorable thermal ranges by the total area in km^2^ of these grid cells and then divided this value by the total area). We also examined future changes in kJ balances within consistently favorable thermal habitats across the North Pacific, to highlight changes in foraging seascapes independent of habitat gain or loss from warming.

### Comparing sources of error in the modeling framework

Linking multiple sub-models together can result in substantial propagation of uncertainty (e.g., [55]). To highlight the contribution of each predictive model to overall uncertainty, we re-ran the modeling framework using 10 simulations each from the posterior distributions of each GAM. This involves extracting the fitted model coefficients and their covariance matrices, simulating new coefficients, and then building new models using these coefficients. This resulted in an ensemble of 10,000 model predictions (10 × 10 × 10 × 10) per earth system model. We compared projections of future kJ balance anomalies for two areas of interest showing relatively strong future change. By comparing the relative contribution of each biological model to overall uncertainty, we can highlight areas where additional observations, or better knowledge of key processes, could reduce uncertainty in future.

## Results

**Fig. 2:**
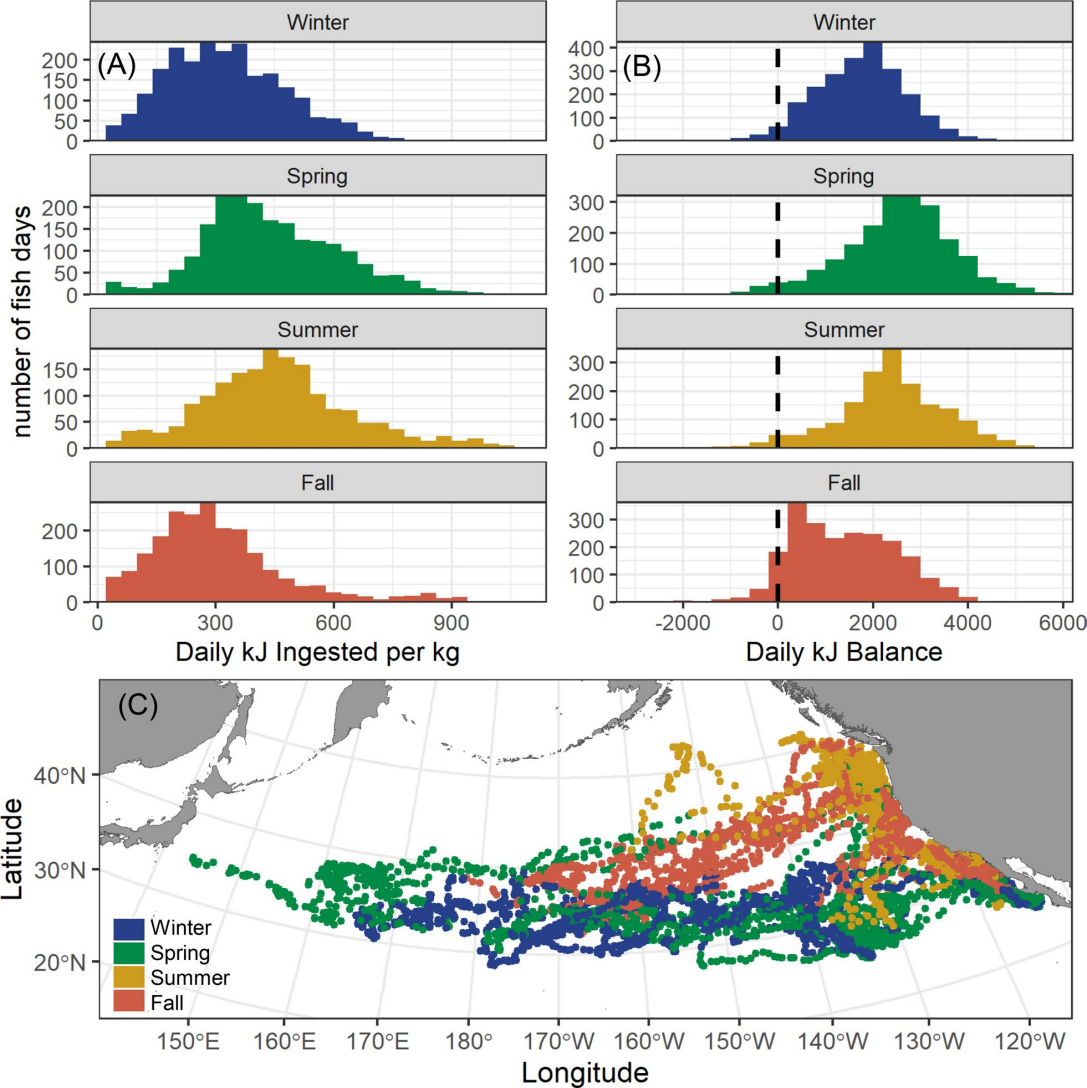
**A** Seasonal histograms of daily kJ ingested per kg body weight for all tagged juvenile albacore predicted using model 4 (Fig. 1, Table 1). **B** Seasonal histograms of daily kJ balance for all tagged juvenile albacore predicted using models 2 and 4 (Fig. 1, Table 1). The vertical dashed lines show a balance of zero. **C** Estimated daily locations of all tagged juvenile albacore

Archival tagged albacore showed extensive seasonal migrations across the North Pacific, ranging thousands of kilometers from the California Current ecosystem to the Kuroshio Current system (Fig. 2).

Fish were generally concentrated along the North American west coast during summer. Some individuals moved offshore in fall, spending winter and spring in the central North Pacific before moving eastwards back towards the California Current system (Fig. 2). Albacore were estimated to consume a median of 356.1 kJ kg^−1^ day^−1^, with higher values in spring (417.4 ± 4.71) and summer (449.5 ± 5.69) versus fall (278.9 ± 4.66) and winter (313.6 ± 3.90). When metabolic costs of movement were accounted for (Table 1, Fig. 1), estimated daily kJ balances were mostly > 0, with the highest median values in spring (2606.9 ± 32.5 kJ) and the lowest values in fall (1301.8 ± 29.1 kJ) (Fig. 2).

**Fig. 3:**
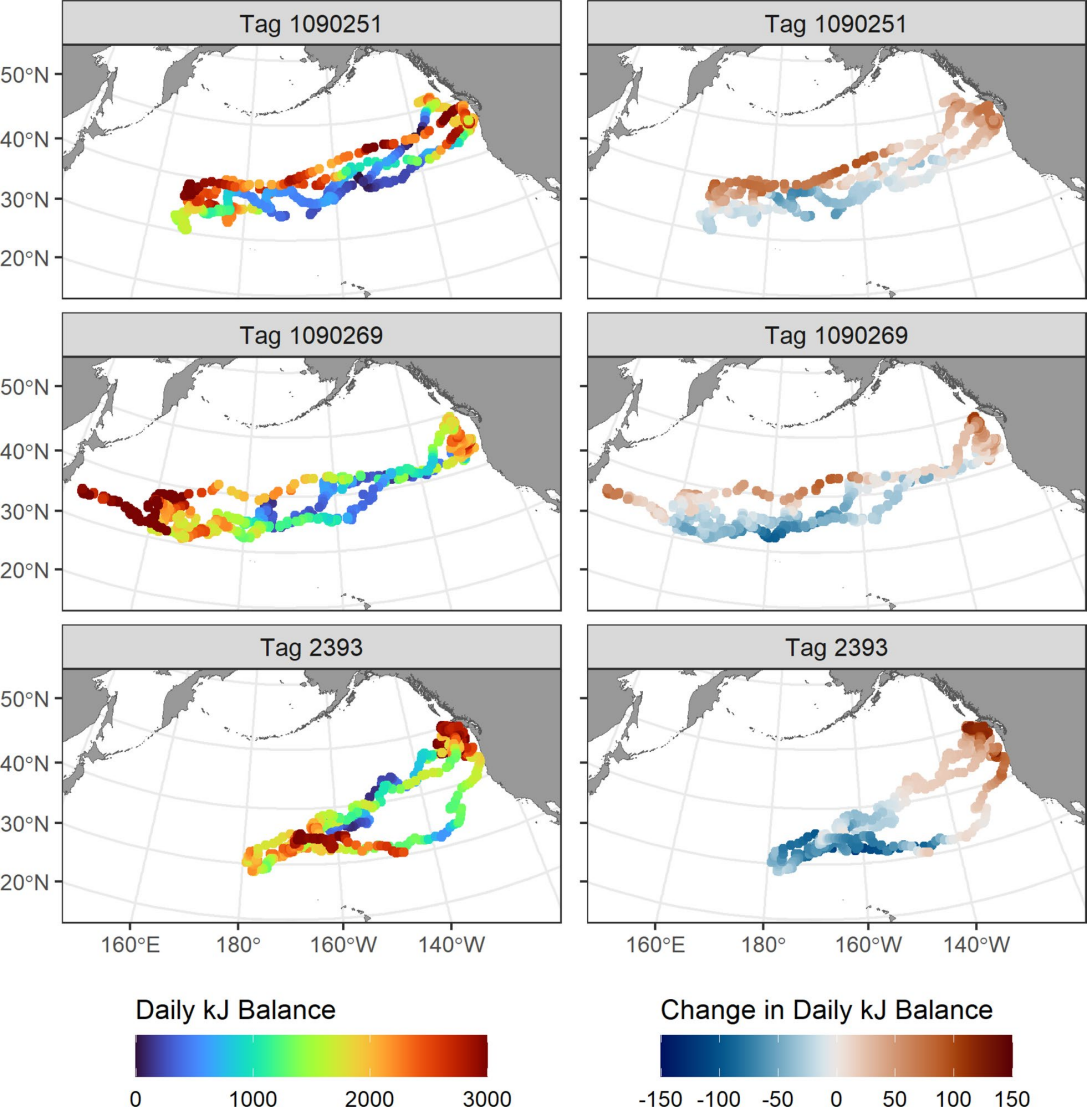
Left: Daily locations and observed kJ balances (7-day running means) along the tracks of three tagged albacore. Right: projected change in kJ balances along the same tracks using 7-day mean kJ balances from historical (1971–2000) versus future (2071–2100) environmental conditions across an ensemble of five earth system models, and observed daily swimming speeds. Tag location data were available for fish 1090251 from September 2nd 2011 to August 23rd 2013, for fish 1090269 from September 3rd 2011 to June 24th 2013, and for fish 2393 from July 30th 2004 to May 26th 2006

To show how projected changes in energetic seascapes could be experienced along observed albacore movement paths, we calculated the change in kJ balance between the historical (1971–2000) and future (2071–2100) periods at the daily locations of three tagged albacore with relatively long tag deployments (Fig. 3).

If these fish followed the exact same routes in both time periods, changes in environmental conditions would result in relatively higher kJ balances in the northern Transition Zone region and the northern California Current. Energetic conditions became generally less favorable in the offshore North Pacific south of ~ 35°N (mean decrease in daily kJ balance of between 19.9 and 52.9 kJ across the three fish). Across the deployment period for each fish however (660–721 days), the mean daily kJ balance did not change substantially. It increased from 1738.3 to 1759.5 kJ daily (21.2 kJ change) between the historical and future time periods at locations occupied by fish 1090251, and remained nearly the same for fish 1090269 and 2393 (decreasing from 1604.2 to 1603.4, and 1671.0 to 1667.9, a change of 0.8 and 3.1 kJ respectively).

**Fig. 4:**
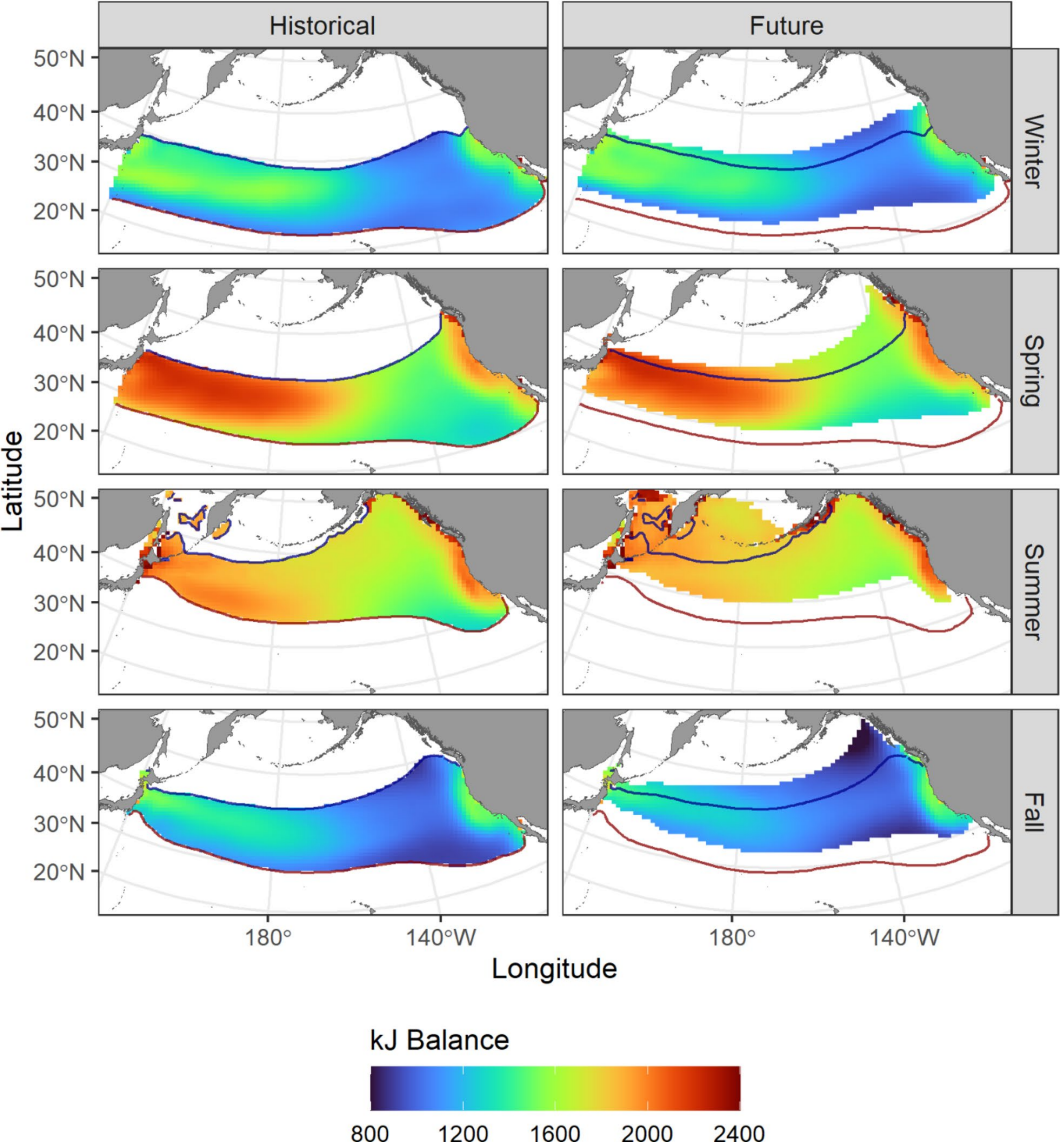
Historical (1971–2000) and future (2071–2100) predicted kJ balances averaged across an ensemble of five earth system models by season. Areas where SST is outside albacore favorable thermal habitat (11–22 °C) are masked. The blue and red contours show the 11 and 22 °C isotherms, respectively, for the historical period, to highlight projected poleward shifts in favorable thermal habitat in future

Broadening application of the modeling framework to the whole North Pacific highlighted substantial seasonality in predicted kJ balance averaged across the historical (1971–2000) earth system model ensemble (Fig. 4).

The highest kJ balance values were along the North American west coast and in the western-central North Pacific in spring and summer, consistent with predictions from individual tagged fish (Fig. 2, Fig. 3). kJ balances were predicted to be lowest in winter and fall between 160 and 140°W. Future Projected future kJ balances showed reasonably similar spatial patterns to the historical period in winter and fall, with some small increases in the western North Pacific near Japan (Fig. 4). However, future kJ balances in summer were more distinct from the historical period, primarily as a result of shifting thermal habitats. With increasing SST, the predicted ideal summer habitat moved northwards into the western Gulf of Alaska and Bering Sea. This poleward shift increased availability of productive subarctic areas with potentially high kJ balances (Fig. 4).

**Fig. 5:**
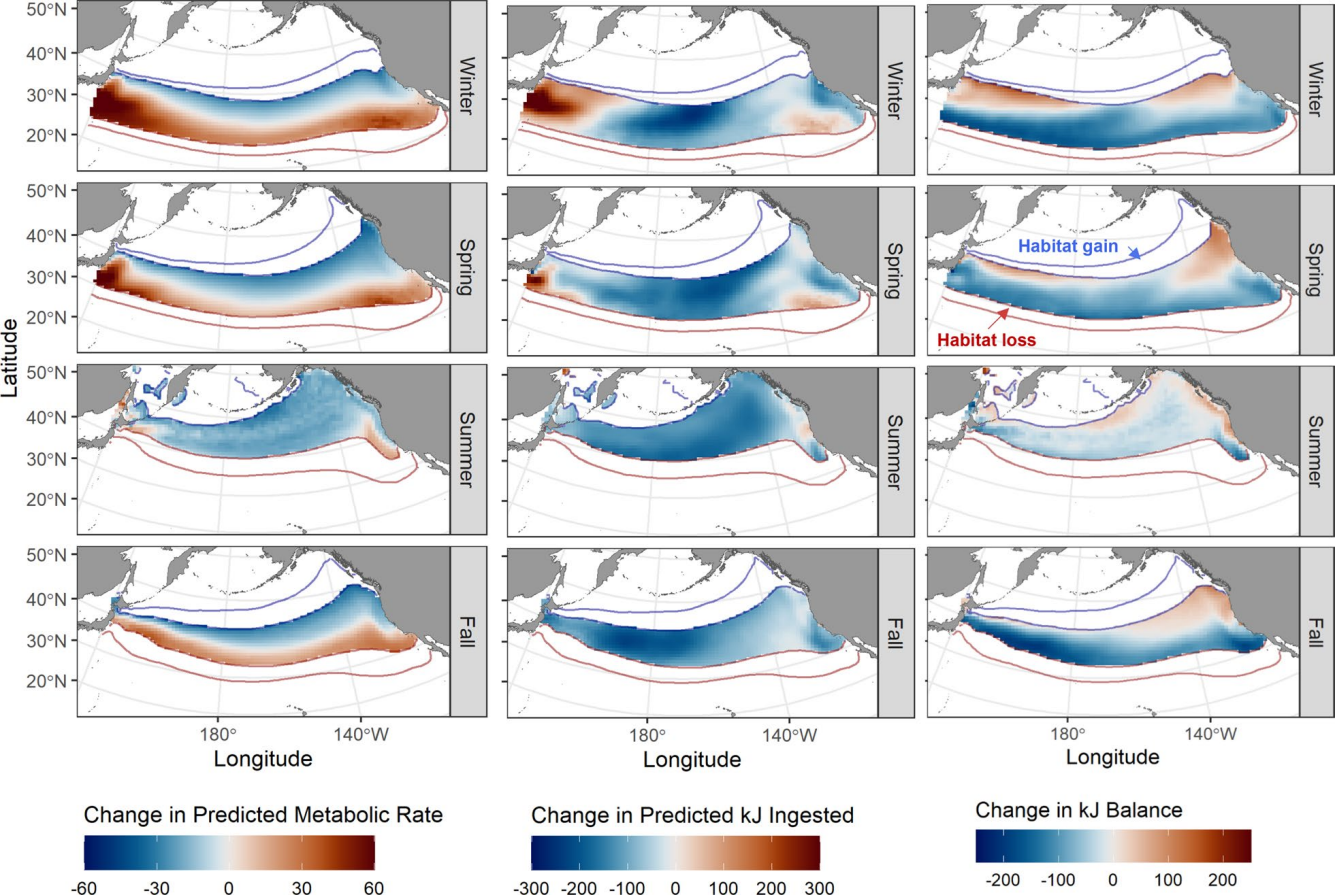
Projected changes to daily metabolic costs of movement (left, mg O_2_ kg-1 h-1), kJ ingested (center), and kJ balance (right). Areas where SST is outside albacore favorable thermal habitat (11–22 °C) are masked. The red lines show the historical and future locations of the 22 °C isotherm, thus areas outlined in red show where future warming results in loss of albacore favorable thermal habitat. The blue lines show the historical and future locations of the 11 °C isotherm, thus areas outlined in blue show where future warming results in gain of albacore favorable thermal habitat. Changes in predicted metabolic rates, kJ ingested and the change in kJ balance are indicated as colored shading in the areas where habitat was thermally suitable in both the past and future time periods

Comparison of predicted energetic costs and gains between the historical and future time periods highlighted considerable spatial heterogeneity. Warming temperatures drove increases in metabolic costs in the southern portion of albacore thermal habitat in winter, spring, and fall, with decreasing costs in the northern part of the species range (Fig. 5, Supplementary Figs. 8–12).

In contrast, predictions of kJ ingested showed stronger longitudinal patterns, with increases in potential energy gain in the western North Pacific in winter and spring, and slight decreases elsewhere. In sum, these changes resulted in potential increases in kJ balance at the northern edge of favorable albacore habitat and decreases in the south. However, the overall negative changes in predicted energy gains and kJ balance did not account for new foraging habitat becoming available as much of the subarctic North Pacific warms to > 11 °C during summer (Fig. 5).

**Fig. 6:**
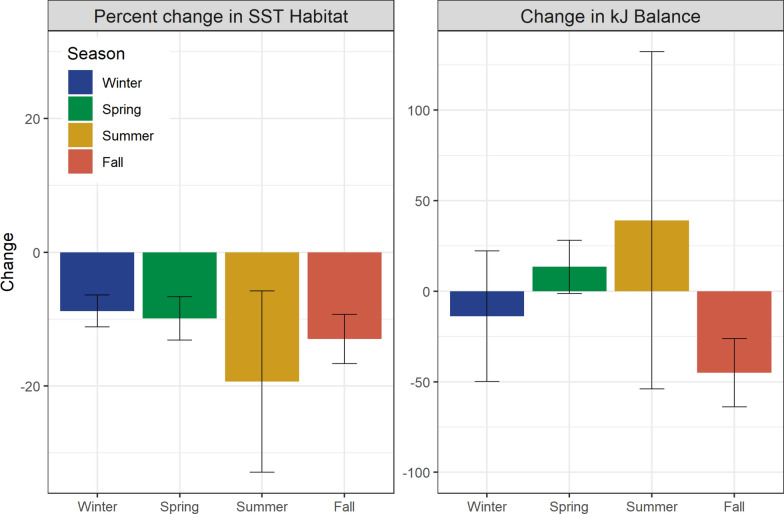
Projected seasonal albacore habitat gain and loss from two methods. Left: change (2071–2100 versus 1971–2000) in thermally favorable habitat defined as 11 °C < SST < 22 °C. Percentage change in habitat area (km^2^) compared to the historical period is shown. Right: change in mean daily kJ balance within thermally favorable habitats. Uncertainty bounds correspond to a 95% confidence interval across the 5 different earth system models

Future warming resulted in an overall loss of favorable thermal habitat for albacore in the North Pacific in all seasons (Fig. 6).

Although there was considerable variability across earth system models, all five projected a smaller extent of thermal habitat in future, with mean losses ranging from 1,612,039 (± 236,375.5) km^2^ in winter, or an 8.8% loss of habitat compared to the historical period, to 2,999,758 (± 1,132,289.6) km^2^ in summer, corresponding to a 19.4% loss of habitat (also see Fig. 5, Supplementary Fig. 13). In contrast, mean daily kJ balance within thermally favorable habitat was projected to decrease in fall, and increase in spring and summer. The five earth system models disagreed on the direction of change in the kJ balance in all seasons except fall, with three models (CMCC-ESM2, CNRM-ESM2-1, IPSL-CM6A-LR) showing a slight increase in winter and three (CMCC-ESM2, GFDL-ESM4, UKESM1-0-LL) showing a moderate increase during spring and summer (Fig. 6).

This disagreement was partially related to spatial differences in projected environmental fields across earth system models. Each model was broadly consistent in showing increased kJ balances in the north and decreases in the south in winter, spring, and fall (Supplementary Fig. 13). However, the spatial extent and location of these areas of change differed across earth system models. In contrast, models showed more disagreement in projected patterns during summer, ranging from a broad increase in future kJ balances (CMCC-ESM2) to a broad decrease (GFDL-ESM4, UKESM1-0-LL) (Supplementary Fig. 13).

This substantial disagreement across earth system models was also evident when we re-ran the modeling framework using 10 simulations from each of the four GAMs in Table 1. Although each GAM contributed considerable uncertainty, the largest source of spread in projections of future change was the choice of earth system model (Supplementary Fig. 14). The uncertainty within projections from each earth system model was also substantial in some areas. The 5th and 95th percentile of future anomalies in kJ balance from the 10 simulations of each GAM overlapped with zero at mid-latitudes in all seasons for the UKESM1-0-LL model (shown as an example), particularly in summer (Supplementary Fig. 15). However, the decrease in projected kJ balance in the southern part of the suitable temperature range for albacore in winter, spring, and fall was robust to uncertainty from the GAMs, as was the future increase in kJ balance in the northern part of the suitable temperature range in all seasons except fall. Thus, while the combination of multiple GAMs and multiple earth system models contributed substantial uncertainty, the broad pattern of an increase in kJ balance in the north, and a decrease in the south, was relatively consistent.

## Discussion

Highly migratory species such as tunas move between areas of optimal foraging and energy balance to maximize growth and reproduction output. In particular, movements between foraging areas can allow mobile animals to increase their energy intake by tracking seasonal concentrations of prey [2]. Climate change is driving shifts in the distribution, abundance, and phenology of foraging areas, as well as altering environmental conditions along migratory paths [29]. As a result, historical migratory strategies may no longer result in favorable balances of energetic costs and gains [49]. Here, we examine changing energetic seascapes for juvenile albacore in the North Pacific Ocean. We show that albacore move within thermally defined corridors to access productive seasonal foraging grounds. Climate-driven warming will shift habitats with suitable temperature characteristics for albacore northwards (Fig. 4). Within these thermally favorable habitats, we show that more southern and offshore areas may become less energetically profitable, while northern and coastal areas may become more profitable (Fig. 5). Importantly, increased access to productive sub-arctic areas may offset the loss of oligotrophic sub-tropical habitats (Fig. 6).

### Migration ecology

Juvenile albacore are distributed across much of the North Pacific, occupying habitats spanning highly productive upwelling regions to the oligotrophic sub-tropical gyre [7, 54, 57]. Their broad temperature tolerances result in a large potential swath of favorable thermal habitat [33]. However, albacore are clearly not randomly distributed within waters of suitable temperature, and they can expend considerable energy migrating long distances between foraging areas each year [57]. Our results suggest that their movement strategies enable albacore to access productive foraging grounds in disparate ecosystems, and that their migration timing tends to place them in favorable foraging areas at strategic times. For example, plankton biomass in the California Current is at a maximum in spring and summer [35], while the strength of the offshore North Pacific Transition Zone chlorophyll front peaks in winter and spring [14]. Tagging and fisheries data show that albacore aggregate in these ecosystems within these highly productive seasons [33, 57].

Other migratory pelagic species in the North Pacific also forage in these productive hotspots. Juvenile Pacific bluefin tuna migrate from the western tropical Pacific to the California Current System to forage as juveniles [34]. Blue whales (*Balaenoptera musculus*) migrate latitudinally between Central America and the Gulf of Alaska, and may time their movements to coincide with times of high productivity in the California Current System [1, 13]. Northern elephant seals (*Mirounga angustirostris*) migrate seasonally between breeding areas along the North American west coast and foraging areas as distant as the western Aleutian Islands [48]. While these top predators use different migration strategies and forage on different prey [7, 68], they consistently visit key foraging areas such as the California Current and the North Pacific Transition Zone [37]. Similar to results shown here for albacore (e.g., Fig. 3), these long-distance migrants may cross large expanses of less productive habitat to reach seasonal foraging grounds [13]. The precise cues driving routes and timing in migratory animals remain incompletely understood. However, if these behaviors evolved based on historical ocean conditions and seasonal cycles, they may become less effective as climate change shifts the mean state and phenology of ocean ecosystems.

### Climate change impacts

Over the coming decades, climate change will push many marine ecosystems into novel states [73]. Key foraging habitats may experience shifts in productivity, seasonal cycles, and prey composition [63]. If, for example, ocean conditions at the start and end points of historical migration routes change at different rates, then migration cues and movement patterns that were historically energetically beneficial may no longer be so [65]. The ability of migratory populations to keep pace with climate change may depend on whether they can modify their behavior to maintain their energetic needs, either through behavioral plasticity or genetic adaptation [5].

Our results show that climate warming in the North Pacific is likely to result in expanded availability of habitat for albacore in the Gulf of Alaska, Bering Sea, and other sub-arctic ecosystems (Fig. 4). These are highly productive systems during the boreal spring and summer [51], and so represent a potentially valuable foraging resource. A key finding is that the potential energetic gains available from enhanced access to the productive sub-arctic may offset losses from the sub-tropics, even with a net loss of habitat area (Fig. 6). Thus, it is clear that examining species distributions using temperature alone does not fully capture the mechanisms driving habitat use. By accounting for the potential biomass of prey contained within areas that become newly favorable or newly unfavorable in future, we can better anticipate both the physiological and ecological consequences of climate change.

Juvenile albacore exhibit multiple movement patterns in the North Pacific, ranging from highly migratory to largely resident [24, 54, 57], and the persistence of these behavior types at a population level varies through time [32]. Albacore may also shift their distributions polewards during unusually warm conditions [25]. This plasticity in habitat use in response to changing ocean conditions has also been observed in other highly migratory pelagic fishes, such as Atlantic bluefin tuna (*T. thynnus*: [41]). Thus even as climate change drives spatiotemporal shifts in North Pacific pelagic habitats and foraging grounds, albacore may have the ability to behaviorally adapt.

### Modeling implications

Previous studies modeling climate change impacts on pelagic predators have used a variety of environmental variables to delineate favorable conditions. Some use only temperature (e.g., [66]) or other physical predictors (e.g., temperature, salinity, mixed layer depth: [50, 86]). Others include biogeochemical predictors such as chlorophyll, primary productivity, phytoplankton biomass, or dissolved oxygen (e.g., [21, 30]). Theoretically, distribution models including biogeochemistry may define foraging habitats more effectively than physics-only models. However, it may be difficult for purely statistical frameworks to capture habitat use in species that travel large distances across unfavorable habitats to reach favorable foraging or breeding areas. The characteristics of “favorable” habitat defined statistically can be highly variable, depending on whether an animal is migrating, foraging, or reproducing. Daily kJ balances calculated for tagged albacore in this study show that they can undergo days to weeks of relatively unfavorable conditions while migrating to areas that are energetically more favorable.

More complicated mechanistic ecosystem models are also available for projecting distributions of pelagic fishes based on changes to physical conditions and prey fields (e.g., [27, 59]). As projections from ecosystem models including higher trophic levels become more available through programs such as FishMIP [10], there are increasing opportunities to move beyond simple correlative distribution models. Existing detailed bioenergetics models for migratory species (e.g., [22]) could also be made more environmentally explicit, and applied to projected environmental fields or multispecies frameworks. However, all such models must include accurate parameterizations of many complex processes in order to provide realistic outputs. As climate change increasingly results in novel ecosystem conditions, achieving accurate parameterization of ecosystem models, skill across trophic levels, and stationarity in species-environment relationships will be particularly challenging. There is thus a clear need to better understand the mechanisms underlying divergent species’ responses to changing conditions [18]. In particular, our results emphasize the importance of direct observations of animals, via laboratory and field studies of metabolism, cost of locomotion, and energetics. When combined with spatiotemporal locations and foraging data, these data provide important new information for parameterizing mechanistic climate impact models.

### Societal impacts

A key motivation for studies of distribution shifts in tunas is to develop scenarios of ecological, economic and social impacts resulting from changing fishing opportunities (e.g., [8]). Over the past several decades, fisheries for juvenile albacore in the eastern North Pacific have experienced large shifts in fishing grounds corresponding to shifts in albacore distribution [32]. Tagging data show that albacore can move rapidly between the exclusive economic zones of the US, Canada, Mexico, and Japan, as well as the high seas, and the proportion of catch in each of these areas has been highly variable historically [32, 57]. Our results show potential future shifts in foraging seascapes for albacore in the North Pacific, with coastal regions potentially becoming more favorable than the high seas. Model outputs can thus inform development of potential future scenarios, and guide equitable decision-making on allocations between evolving and declining fisheries. However, our analyses highlight that variability across earth system model projections is a large source of uncertainty when developing modeling frameworks, and may preclude the development of scenarios at high spatiotemporal resolution. In addition, it is difficult to anticipate how fishing fleets and communities will respond in the context of complex international management agreements, and variable responses of individual vessels and fleets. In the US, the fishing fleet that targets juvenile albacore is heterogeneous, composed of different-sized vessels with different characteristics, fishing strategies, and fishery portfolios [32]. Each vessel thus has a differing ability to follow shifting distributions of fish, or to switch to targeting other species based on markets and availability. Future studies of climate change impacts on North Pacific albacore will need to more fully incorporate human dimensions and socioeconomic scenarios in order to provide actionable advice to natural resource managers.

### Knowledge gaps and future work

Our study framework and results highlighted several important knowledge gaps, and opportunities for future research. A key uncertainty was the lack of laboratory data on albacore, as they are difficult to keep and study in captivity. While Pacific bluefin tuna are closely related to albacore and have similar thermal preferences, they have different migratory behaviors and grow to a much larger size. A lack of physiological observations is a common issue when developing mechanistic ecological models, particularly for larger species or those which do not survive captivity. As in this study, validating the realism of models using a wide variety of field and laboratory observations may be the best approach for addressing uncertainty in physiological responses.

As the cues driving migratory behavior in albacore are incompletely understood, it is difficult to anticipate how they will respond to longer term climate change. Migratory behaviors can be learned, innate, or a combination of the two [3]. Climate change may impact environmental migration cues (e.g., temperature) directly, while geographic cues (e.g., magnetic fields) stay within historical bounds [49]. The short time-period (2003–2013) covered by our tagging data also limited our ability to ground-truth our projections using historical variability (e.g., marine heatwaves, [78]). As our tags were archival, we also have very little information on finer-scale movements. We thus used fixed diel swimming speeds in our framework, which will highly oversimplify energetic costs of movement. Newer electronic tags with more physiological sensors may offer opportunities to further develop mechanistic models in future.

While the use of multiple GAMs contributed substantial uncertainty to our modeling framework, the largest source of uncertainty was divergence across earth system models. Including an ensemble of climate models is essential for capturing and communicating this uncertainty. Mesozooplankton biomass projections vary widely across earth system models, as a result of different biogeochemical sub-models and a lack of observations to parameterize and validate them [61]. Due to the size of our study domain, we also did not downscale the earth system models. Finer-scale processes in coastal upwelling systems such as the California Current system were thus likely not well resolved [63]. For example, coastal temperature fronts are known to be important for albacore foraging success [75, 85], but these features are not captured by coarse-resolution models. Sub-surface prey biomass on sub-tropical foraging grounds is also not well captured in many biogeochemical models [83]. Using mesozooplankton biomass to estimate albacore prey will not capture changes in forage composition, nutritional quality, or broader foodweb structure, or interacting effects such as species-specific temperature or oxygen tolerances [38, 82]. While substantial work to improve biogeochemical models continues, our results are best interpreted at broad spatiotemporal scales, with close attention paid to where earth system models diverge, and uncertainty is greatest.

In addition, predators show flexible foraging behavior in response to prey availability and ocean conditions. Albacore show different vertical movement patterns in coastal versus offshore waters as they target prey layers at different depths [7, 57]. They can also rapidly switch between prey types (e.g., finfish versus cephalopods) in response to availability [58]. This type of adaptive foraging ecology is difficult to capture in models. However, assuming fixed trophic connections may underestimate the potential of predators to adapt to future change.

## Conclusions

Overall, we show that juvenile albacore move substantial distances within favorable thermal habitat to forage in seasonally productive ecosystems in the North Pacific. Future climate change may result in loss of favorable thermal habitats in the sub-tropics, but allow increased access to productive sub-arctic ecosystems during summer. Coastal ecosystems may become more energetically favorable in future, while the offshore North Pacific becomes less favorable, largely as a result of lower potential energetic gains from foraging. The largest contributor to uncertainty within our framework was variability among earth system models, which highlights the importance of using multi-model ensembles for climate change impact studies. Despite the uncertainty introduced by including biogeochemistry in our framework, we clearly show the importance of moving beyond temperature and considering energetics when assessing climate change impacts on marine ecosystems. Further work is needed to better understand how highly migratory animals use movement behaviors to optimize energetic gains, and how climate change will influence foraging seascapes and energetic tradeoffs.

## Supplementary Information

Supplementary material 1

## Data Availability

Albacore tracks from the TOPP program are available through the TOPP data interface (https://gtopp.org/). Additional data from tags available through the Albacore Archival Tagging Program are not posted publicly at the request of the American Fishermens Research Foundation, but are available upon request to BM. The mesozooplankton biomass fields are described in Park et al. [60], and available upon request to J-YP. Earth system model projections are freely available from the Earth System Grid Federation (aims2.llnl.gov).
