# Supplementary material for: Climate change impacts to foraging seascapes for a highly migratory top predator
Source: Mov Ecol. 2025 May 9;13:33. doi: 10.1186/s40462-025-00558-1 (PMC12065262; doi:10.1186/s40462-025-00558-1)
Supplement: Supplementary file 1 — Supplementary material 1 [file 40462_2025_558_MOESM1_ESM.pdf]

Supplementary Materials

Supplementary Methods

Predictive Models (GAMs)

All models used a Gamma distribution with a log-link, except model 3 which used a Gaussian distribution. Acceptable fit of each model was assessed using scatterplots of residuals, Q-Q plots, and examination of the autocorrelation function of the residuals where appropriate. The maximum number of knots (k) was constrained (usually to k = 3 or k = 4) to prevent biologically unrealistic partial responses where necessary.

Field Observations

Details of the two albacore archival tagging programs, and processing of data from recovered tags, are described in Muhling et al. (2022), with a summary provided here. We use data from 28 recovered tags with > 30 days of available data. These albacore were released off the U.S. West Coast and Baja California at between 63.2 cm and 96.2 cm straight fork length, between July 2003 and August 2011, and spent between 43 and 752 days at large. Daily light-based geolocations were refined using first a sea surface temperature (SST) inclusive unscented Kalman filter (Lam et al., 2008; Neilsen et al. 2012), and then a bathymetric correction constraining estimated locations based on daily fish depth (Galuardi et al., 2010).

The archival tags fitted to juvenile albacore recorded both internal body temperatures and external ambient temperatures every minute, allowing the calculation of daily Heat Increment of Feeding (HIF: see below) integrated across a 24-hour time-period for each fish. Each animal's baseline (fasting) body temperature was estimated using night-time records, when albacore typically do not feed (see Muhling et al. 2022), and stay in the upper mixed layer within relatively constant water temperatures. During the night, albacore body temperature slowly declines as prey captured during the day are digested, reaching a minimum just before dawn. HIF was thus calculated as the area under the curve between the estimated baseline body temperature and observed body temperature at 1-minute intervals, across each 24-hour period (for more details on HIF calculation see Muhling et al. 2022).

Length (cm fork length) was estimated assuming linear growth between the recorded lengths at release and recapture, and checked for consistency with published otolith growth rates (Xu et al. 2016). Where no recapture length was recorded (n = 17 fish), we used mean growth rates from the fish where both measurements were available.

We predicted the daytime and nighttime depth of tagged albacore (models 1a and 1b) using environmental conditions. When conditions are warmer or more oxygenated, fish forage at deeper depths during the day. During the night, they remain in the upper water column, and their depth is strongly tied to moon phase (Frawley et al. 2024, Matsubara et al. 2024). We investigated predictors capturing vertical foraging conditions (e.g., depth of the chlorophyll maximum), but they did not add any predictive skill to the models, and were excluded.

We hypothesized that HIF would be correlated with prey concentrations, and that these would be somewhat predictable based on biomass of lower trophic levels. Mesozooplankton biomass was therefore matched to the locations of tagged albacore as a proxy for food availability (see Muhling et al. 2022). This field was obtained at monthly 3x3 degree resolution from a data-assimilative retrospective physical ocean simulation integrated with a non-assimilative biogeochemical/plankton food web model (Park et al. 2018). To better match the timescales of biogeochemical model output, and reduce the

temporal autocorrelation issues identified by Muhling et al. (2022), this model (model 3) predicts weekly mean HIF for each tagged fish. Day length was calculated using date and latitude using the *geosphere* package (Hijmans 2022).

Laboratory Observations

We used data from captive Pacific bluefin tuna (*Thunnus orientalis*) to estimate metabolic rates and kJ intake, as there are insufficient measurements available for albacore where fish were not artificially stressed by recent capture (e.g. Graham & Laurs 1982). Bluefin tuna have relatively similar physiology to albacore, and the two species inhabit similar thermal ranges as juveniles (Childers et al. 2011; Fujioka et al. 2018), so these data are likely the closest approximation available for estimating juvenile albacore metabolic rates (see additional details in Muhling et al. 2022).

We estimated metabolic rates from temperature and swimming speed using laboratory data from Blank et al. (2007a, b) and Clark et al. (2013). The bluefin tuna examined in these studies were approximately 8 - 10 kg in weight. Based on estimated daily growth rates and published length-weight relationships for albacore (Chen et al. 2012), tagged albacore were likely between approximately 5 and 25 kg during their time at large.

We therefore adjusted predictions of metabolic rate from the model trained on data from 8 – 10 kg bluefin tuna (which are in mg O_2 kg^{-1} $hour^{-1}$) using the scaling coefficient from Brill (1987):

$SMR = 286.8\ W^{0.573}$

where SMR is standard metabolic rate and W is the weight of the fish. However, we recognize that there is very large uncertainty in scaling coefficients for endothermic animals, and that metabolic rates have not been accurately measured for large tunas (Blank et al. 2007a).

HIF was quantified in captive bluefin tuna by Whitlock et al. (2013). They defined HIF by integrating the area between the observed body temperature and the fasting body temperature, accounting for both the duration and magnitude of the internal temperature increase (Whitlock et al. 2013; 2015). After feeding, the internal body temperature of the fish increases, and stays elevated above their fasting body temperature for several hours. To estimate relationships between energy intake (in kJ) and HIF for this study, we used data from 13.8 – 16.7 kg captive Pacific bluefin tuna (Whitlock et al. 2013). This study showed that HIF is strongly correlated to the caloric value of a meal, and so is a useful estimate of energy intake. However, HIF is also influenced by prey species and ambient temperature. HIF was lower for sardine (*Sardinops sagax*) meals than squid (*Doryteuthis opalescens*) meals of the same caloric value, likely due to differing protein and fat content of the two prey species. HIF was also greater at cooler ambient temperatures (Whitlock et al. 2013). Diet studies from the eastern North Pacific show that albacore diet varies, but is about 70% finfish prey (e.g., sardine, anchovy *Engraulis mordax*, saury *Cololabis saira*), with the rest commonly cephalopods (e.g., *Onychoteuthis borealijaponica, Doryteuthis opalescens*), and other invertebrates such as krill or amphipods. These proportions are relatively constant whether albacore diets were sampled from fish caught in coastal areas or far offshore (Iversen 1962; Glaser et al. 2015; Nickels et al. 2023). Based on records of energy density, lipid content, and protein content in these prey species (Gleiber et al. 2024), using a value of 70% finfish prey for wild albacore likely captures the approximate nutritional composition of their diet.

Projecting future changes in thermal and energetic habitats

Outputs from all earth system models were bilinearly interpolated to a common 3-dimension spatial grid (1x1 degree horizontal resolution, vertical resolution of 2.5m in the upper 20m, 5m from 20 - 50m, 10m

from 50m - 100m, 20m from 100m - 200m, 25m from 200m - 300m, and 25m from 300m – 400m, using Climate Data Operators (Schulzweida 2023). This finer vertical interpolation lessened sharp temperature changes across adjacent predicted fish depths.

We did not attempt to bias-correct the earth system model fields against historical observations. Climate model biases can result from complex interactions of the climate system (Stock et al. 2011), and we did not have sufficient high-quality observations to complete this for the oxygen or mesozooplankton fields (Petrik et al. 2022). However, comparison of SSTs from the NOAA 0.25° Daily Optimum Interpolation Sea Surface Temperature (OISST) product, version 2.1 (Reynolds et al. 2007; Huang et al. 2021) and the historical runs of the 5 earth system models suggests that earth system model ensemble captures the seasonal and spatial shifts in albacore thermal habitats (**Figure 2**) fairly well (**Supplementary Figure 5)**. Our results should therefore be useful at a broader spatial scale covering the major North Pacific Ocean biomes (e.g., the subtropical gyre, North Pacific Transition Zone etc.).

Supplementary Tables

Supplementary Table 1: Details of the five earth system models used for future projections (after Petrik et al. 2022).

Center	Earth system model	Reference	Ecosystem Model	Ecosystem Model Reference
Euro-Mediterranean Center on Climate Change	CMCC-ESM2	Lovato et al. 2022	BFMv5.2	Vichi et al. 2020
National Meteorological Research Centre	CNRM-ESM2-1	Séférian et al. 2019	PISCES 2.0gas	Aumont et al. 2015
NOAA Geophysical Fluid Dynamics Laboratory	GFDL-ESM4	Dunne et al. 2020	COBALTv2	Stock et al. 2020
Institute Pierre-Simon Laplace	IPSL-CM6A-LR	Boucher et al. 2020	PISCES 2.0gas	Aumont et al. 2015
Met Office and National Environment Research Council	UKESM1-0-LL	Sellar et al. 2019	MEDUSA2.1	Yool et al. 2013

Supplementary Figures

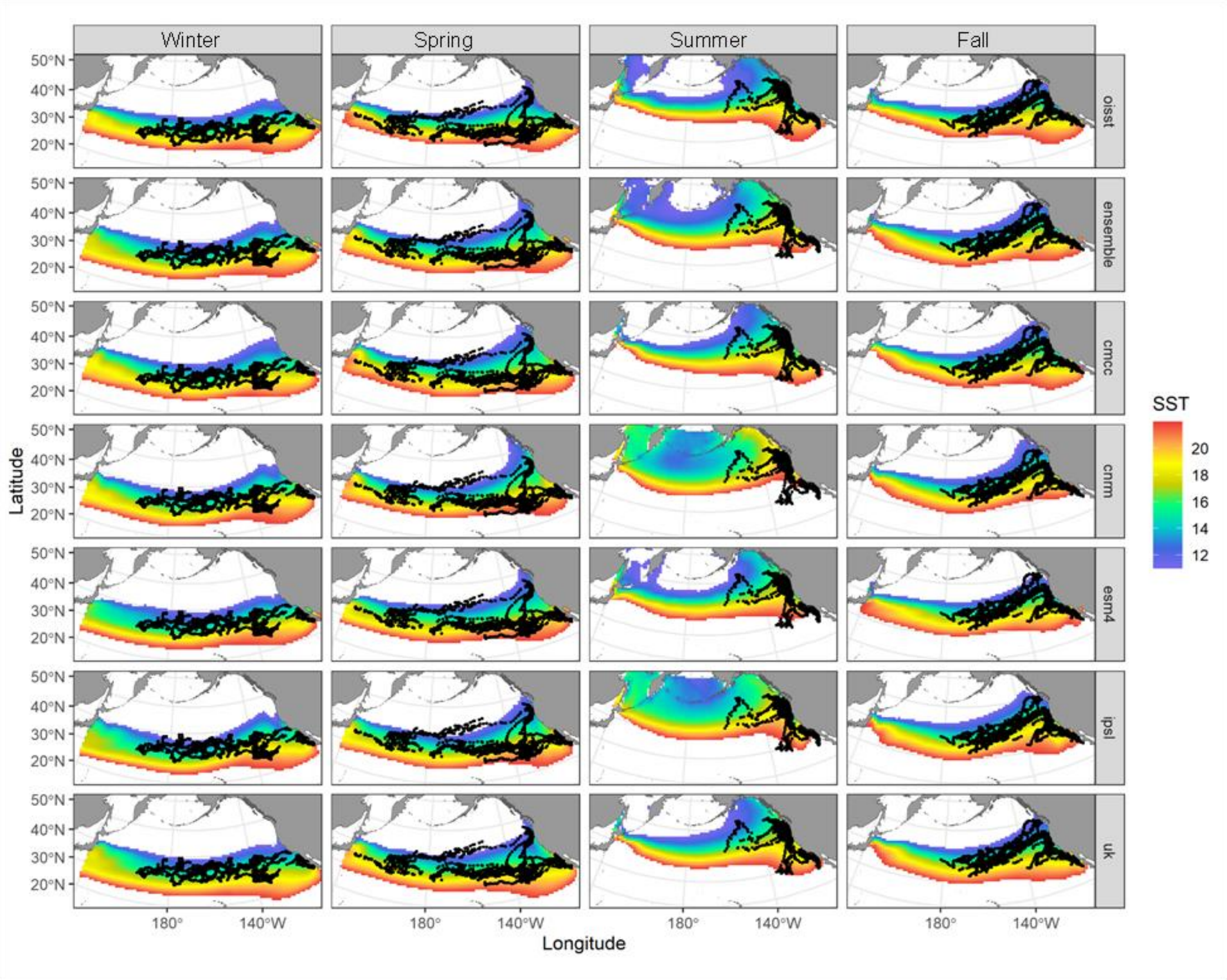

Supplementary Figure 1: Albacore tag locations for years 2003 – 2013 overlaid on sea surface temperature (SST) across seasons (winter is January – March, spring is April – June etc.), showing their overall distribution in the North Pacific Ocean. SSTs are shown as climatologies from observations (OISST: top), from an ensemble of all five earth system models for the historical period 1971 – 2000 (second row), and for each earth system model separately (remaining rows). SSTs outside the favorable range for juvenile albacore (11 – 22°C) are masked.

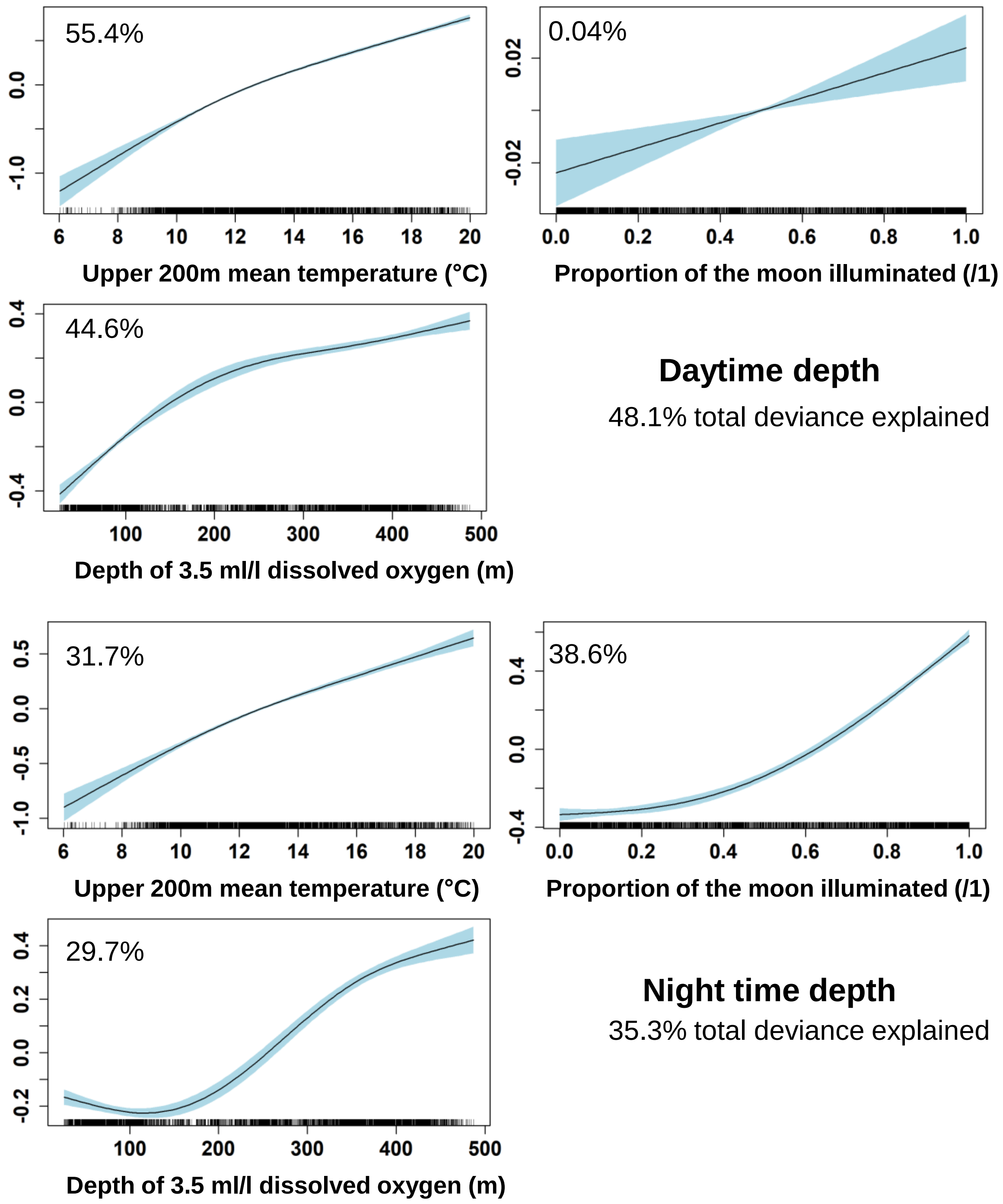

Supplementary Figure 2: Partial response plots for Generalized Additive Models 1a and 1b predicting daytime depth of tagged albacore (top), and night time depth (bottom). Note that a positive partial response indicates an increase with depth (i.e., depth is measured as a positive variable, not negative). The relative importance of each predictor is shown, as a proportion of the total deviance explained (i.e. numbers sum to 100%).

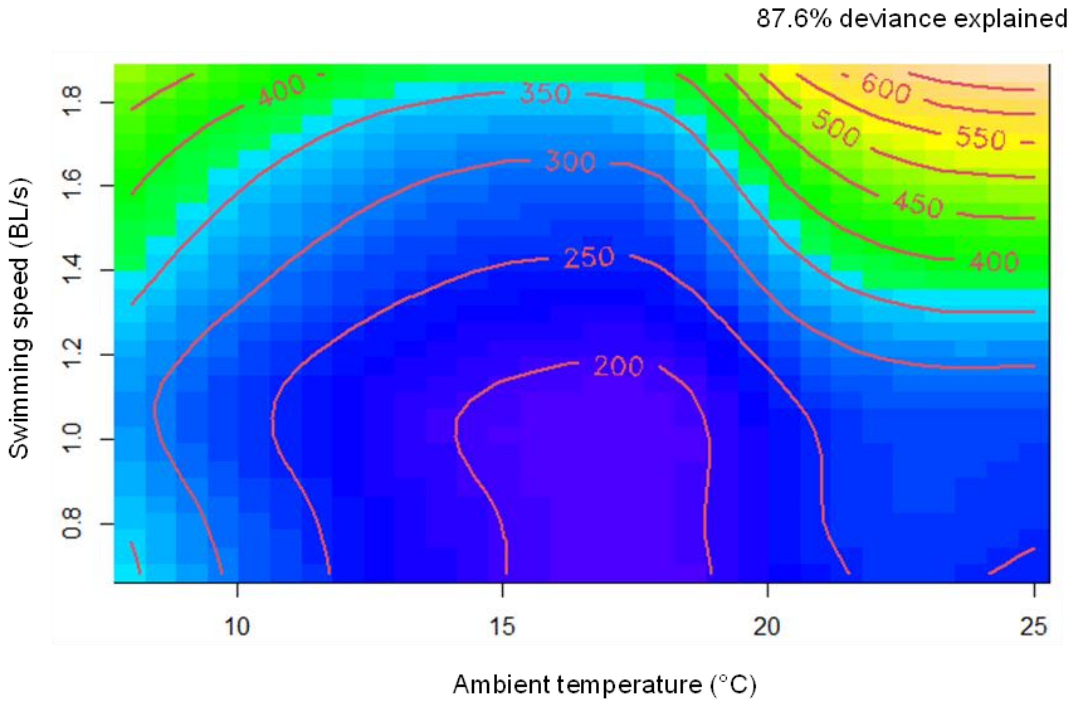

Supplementary Figure 3: Partial response plot for Generalized Additive Model 2 predicting metabolic costs of movement (mg O_2 kg^{-1} hr^{-1}) with respect to ambient temperature (x-axis) and swimming speed in body lengths per second (y-axis).

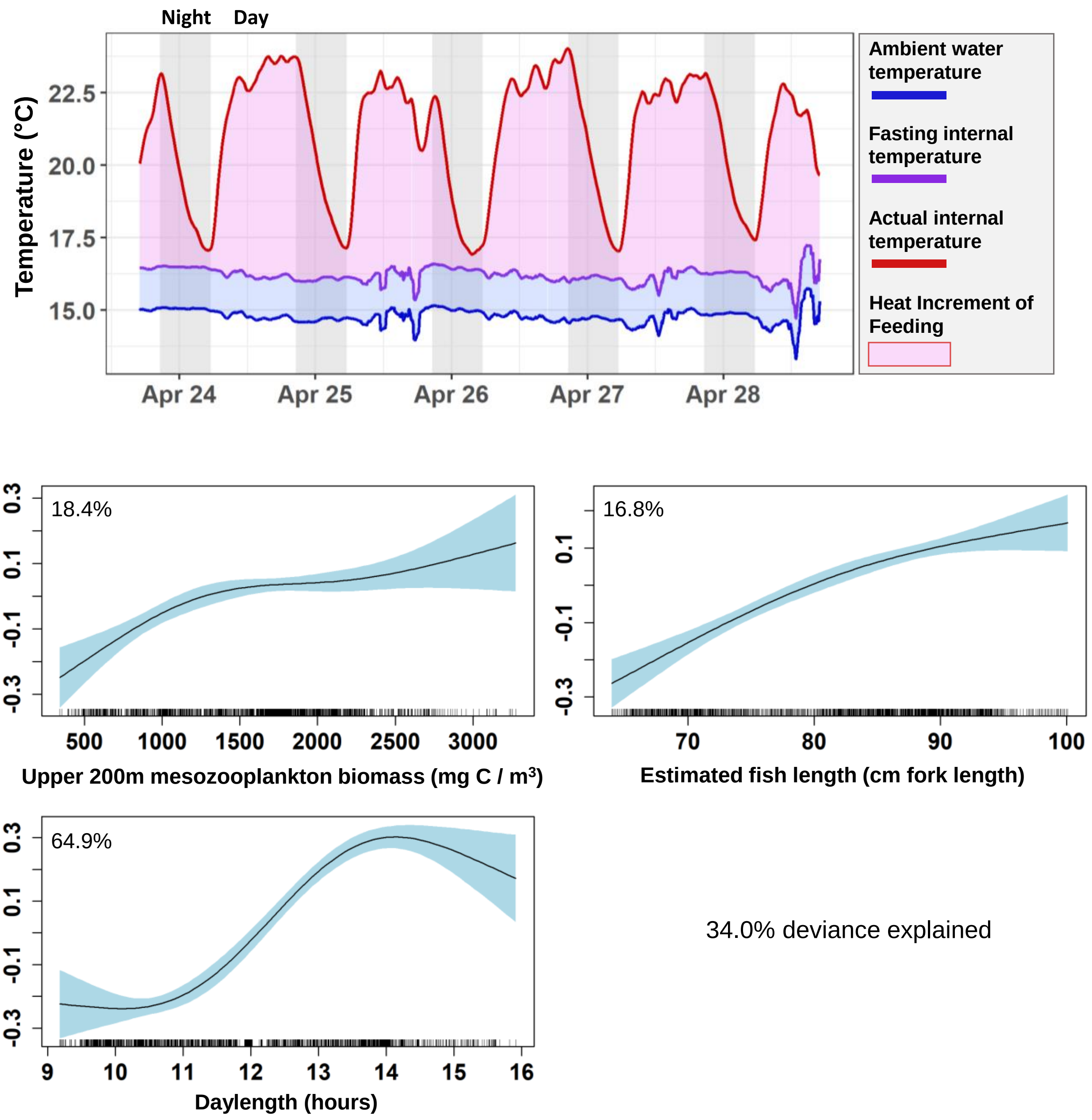

Supplementary Figure 4: Top: Example of Heat Increment of Feeding (HIF) in a tagged albacore, modified from Muhling et al. 2022. Shown are a 1-h moving mean of ambient water temperature (blue), estimated baseline (fasting) internal temperature (purple), and measured internal temperature (red). The light pink area denotes the internal temperature increase due to HIF, while the purple area is the estimated thermal excess maintained by the fish under fasting conditions. Shading shows nighttime (gray) and daytime (white), defined using nautical dusk and nautical dawn. Bottom: Partial response plots for Generalized Additive Model 3 predicting Heat Increment of Feeding (°C hr) with respect to mesozooplankton biomass, fish length, and day length. The relative importance of each predictor is shown, as a proportion of the total deviance explained (i.e. numbers sum to 100%).

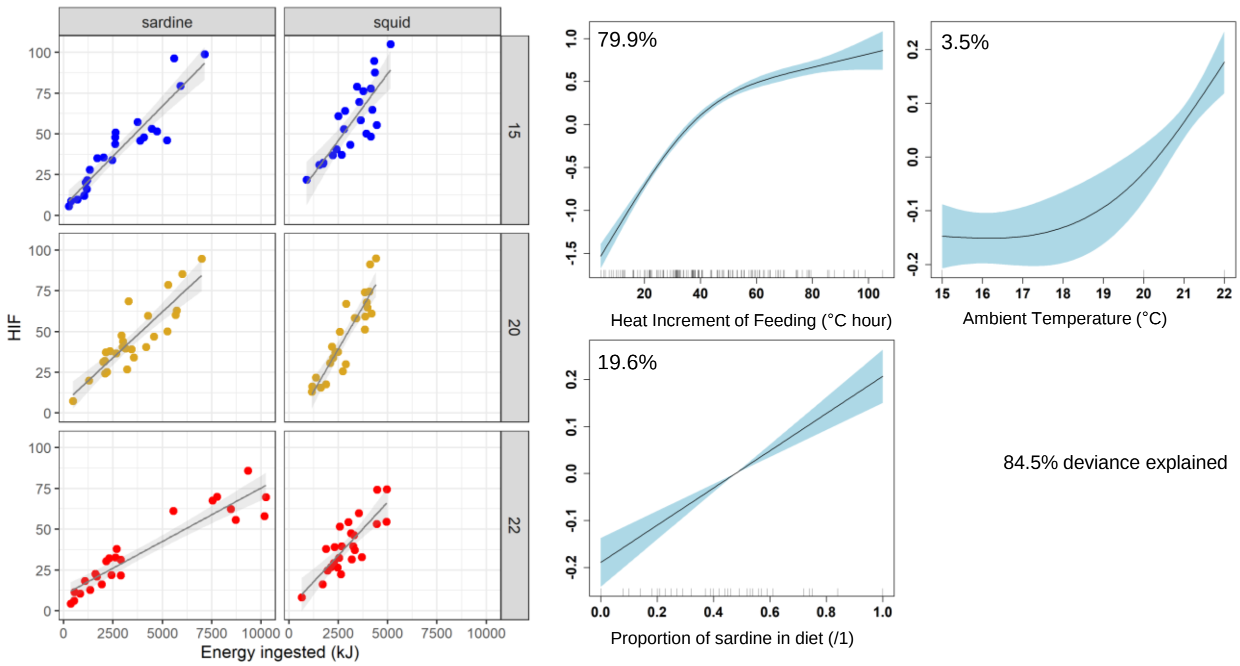

Supplementary Figure 5: Left: observations from Whitlock et al. (2013) showing HIF versus kJ ingested in bluefin tuna for fish fed sardine versus squid and held at different temperatures. Right: Partial response plots for Generalized Additive Model 4 predicting kJ ingested from HIF, ambient temperature, and the proportion of sardine in the diet. The relative importance of each predictor is shown, as a proportion of the total deviance explained (i.e. numbers sum to 100%).

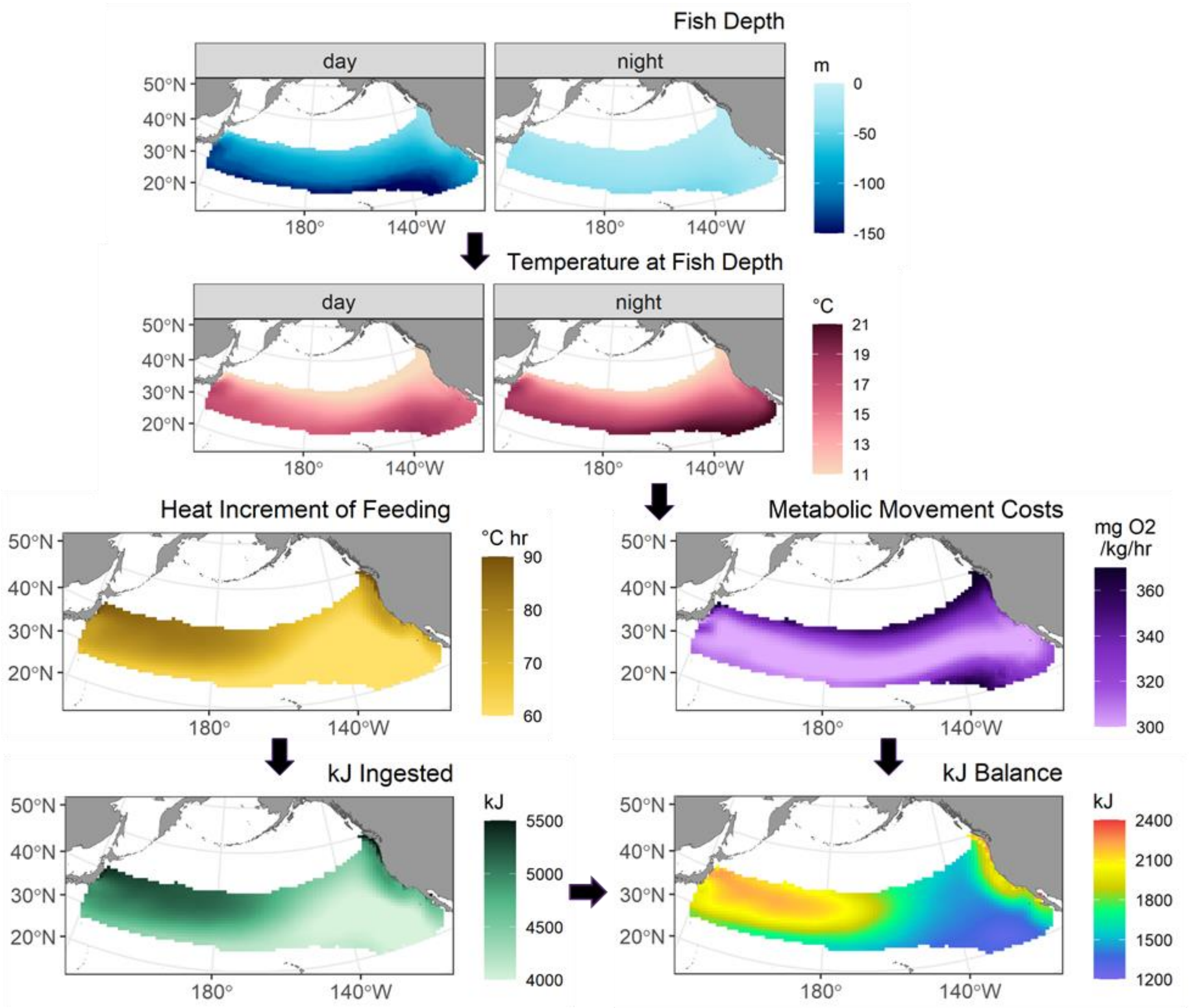

Supplementary Figure 6: Demonstration of the modeling workflow for the historical period (1971 – 2000) in spring (April – June). Maps show means of fields calculated for each earth system model separately. Note that fish depth and temperature at depth are calculated for day and night separately, due to diel vertical behavior patterns in albacore. Other fields are integrated to show a 24-hour daily value. Areas where SST is outside albacore favorable thermal habitat (11 – 22 °C: see **Supplementary Figure 6**) are masked. Maps for all seasons across historical and future time-periods are shown in **Supplementary Figures 8 – 12**.

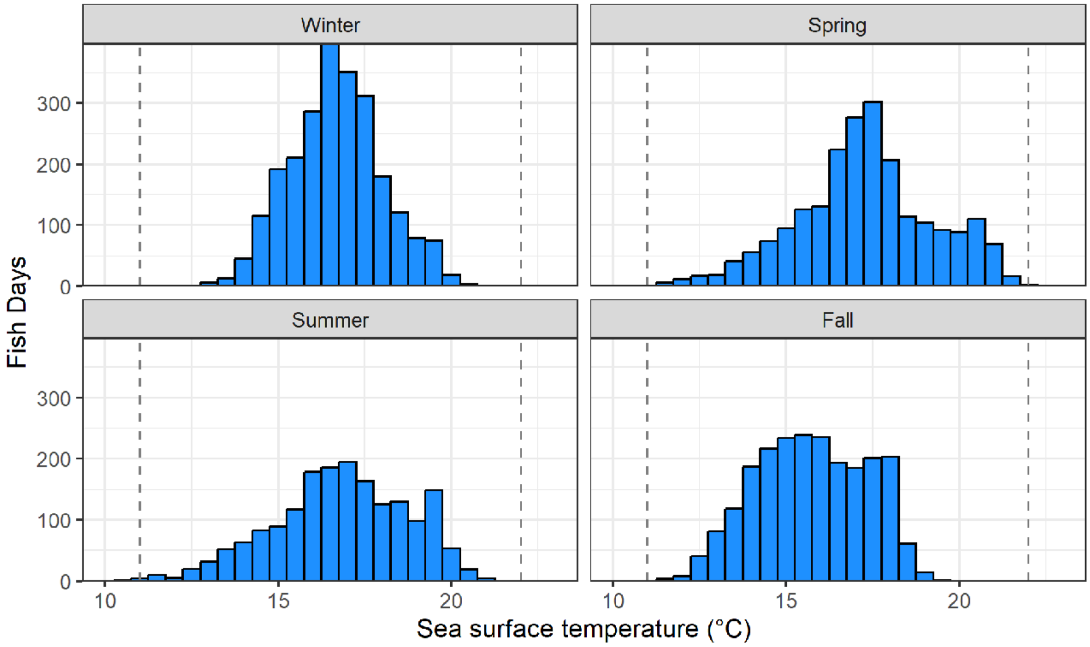

Supplementary Figure 7: Histograms of sea surface temperature from OISST at the estimated daily locations of 28 archivally-tagged albacore at large between 2003 and 2013. Seasons shown are winter (January – March), spring (April – June), summer (July – September) and fall (October – December). Vertical dashed lines show 11°C and 22°C, used as indicators of suitable albacore thermal habitat in subsequent analyses.

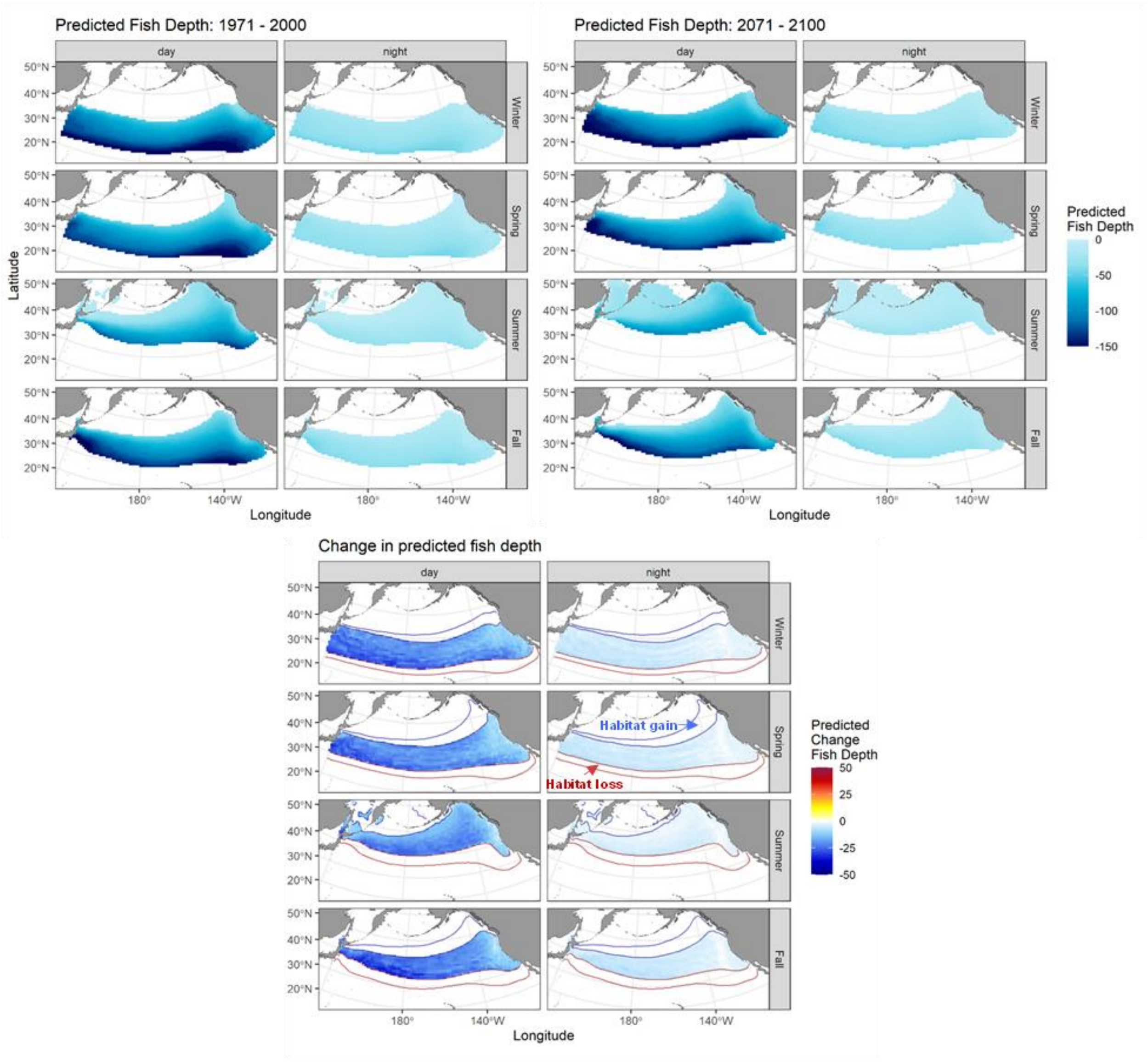

Supplementary Figure 8: Predictions of daytime and night time fish depth for the historical period (top left), future period (top right), and the predicted change between the two time periods (bottom). Areas where SST is outside albacore favorable thermal habitat (11 – 22 °C) are masked. The red lines show the historical and future locations of the 22 °C isotherm, thus areas outlined in red show where future warming results in loss of albacore favorable thermal habitat. The blue lines show the historical and future locations of the 11 °C isotherm, thus areas outlined in blue show where future warming results in gain of albacore favorable thermal habitat.

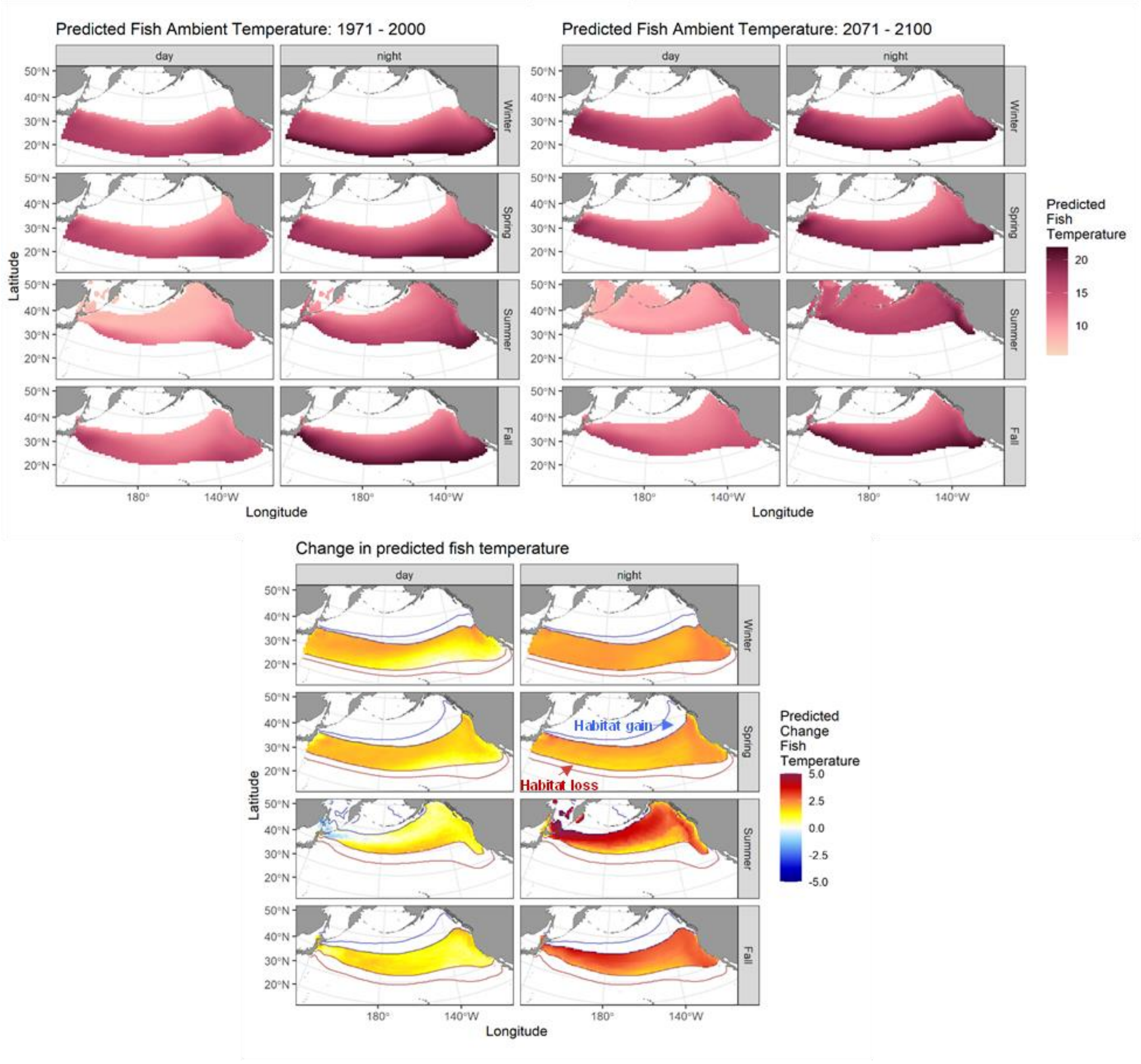

Supplementary Figure 9: Predictions of daytime and night time ambient fish temperature (at predicted fish depth) for the historical period (top left), future period (top right), and the predicted change between the two time periods (bottom). The red lines show the historical and future locations of the 22 °C isotherm, thus areas outlined in red show where future warming results in loss of albacore favorable thermal habitat. The blue lines show the historical and future locations of the 11 °C isotherm, thus areas outlined in blue show where future warming results in gain of albacore favorable thermal habitat.

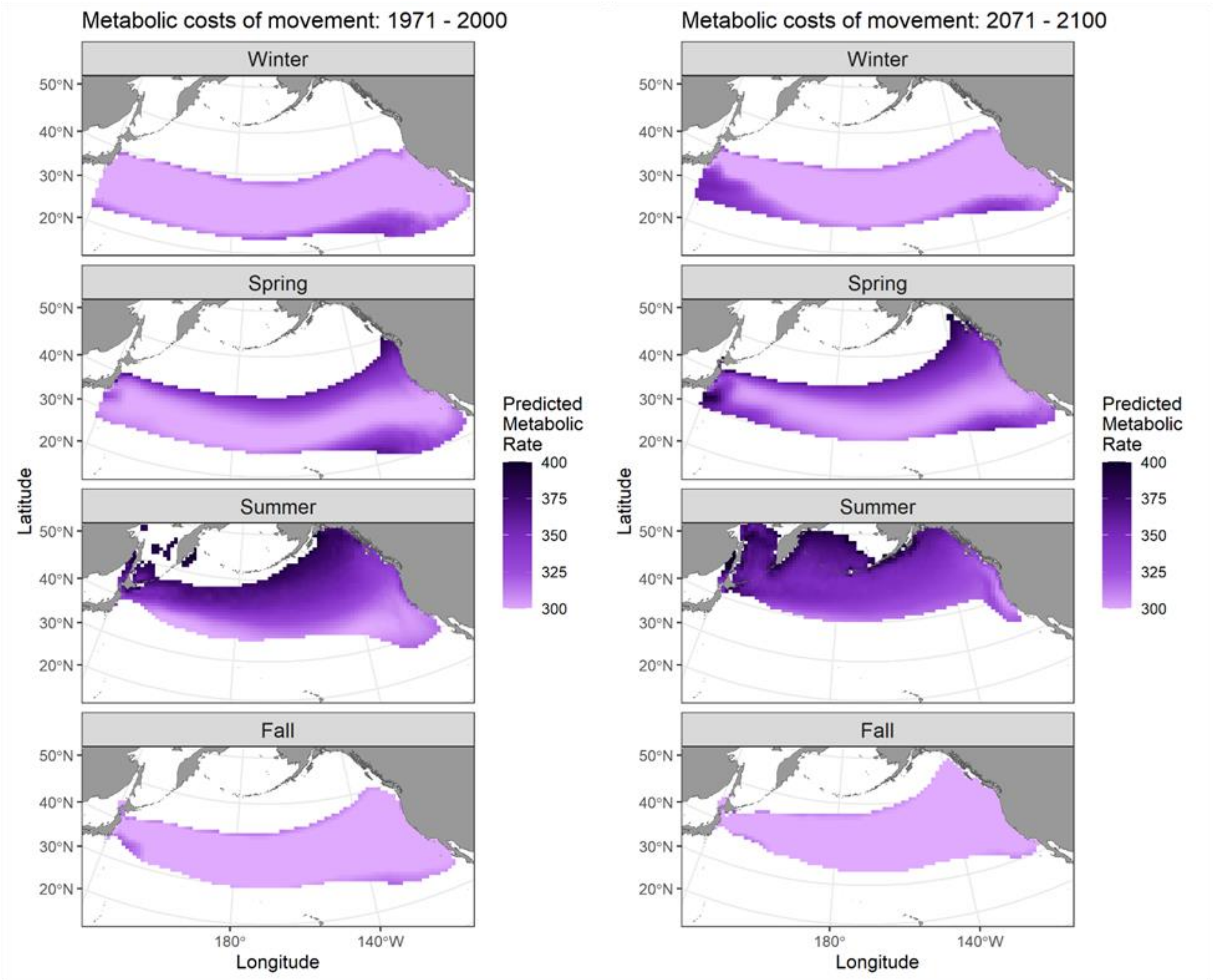

Supplementary Figure 10: Predictions of metabolic costs of movement for the historical period (left) and future period (right). Areas where SST is outside albacore favorable thermal habitat (11 – 22 °C) are masked. Future anomalies are shown in the main text (Figure 6).

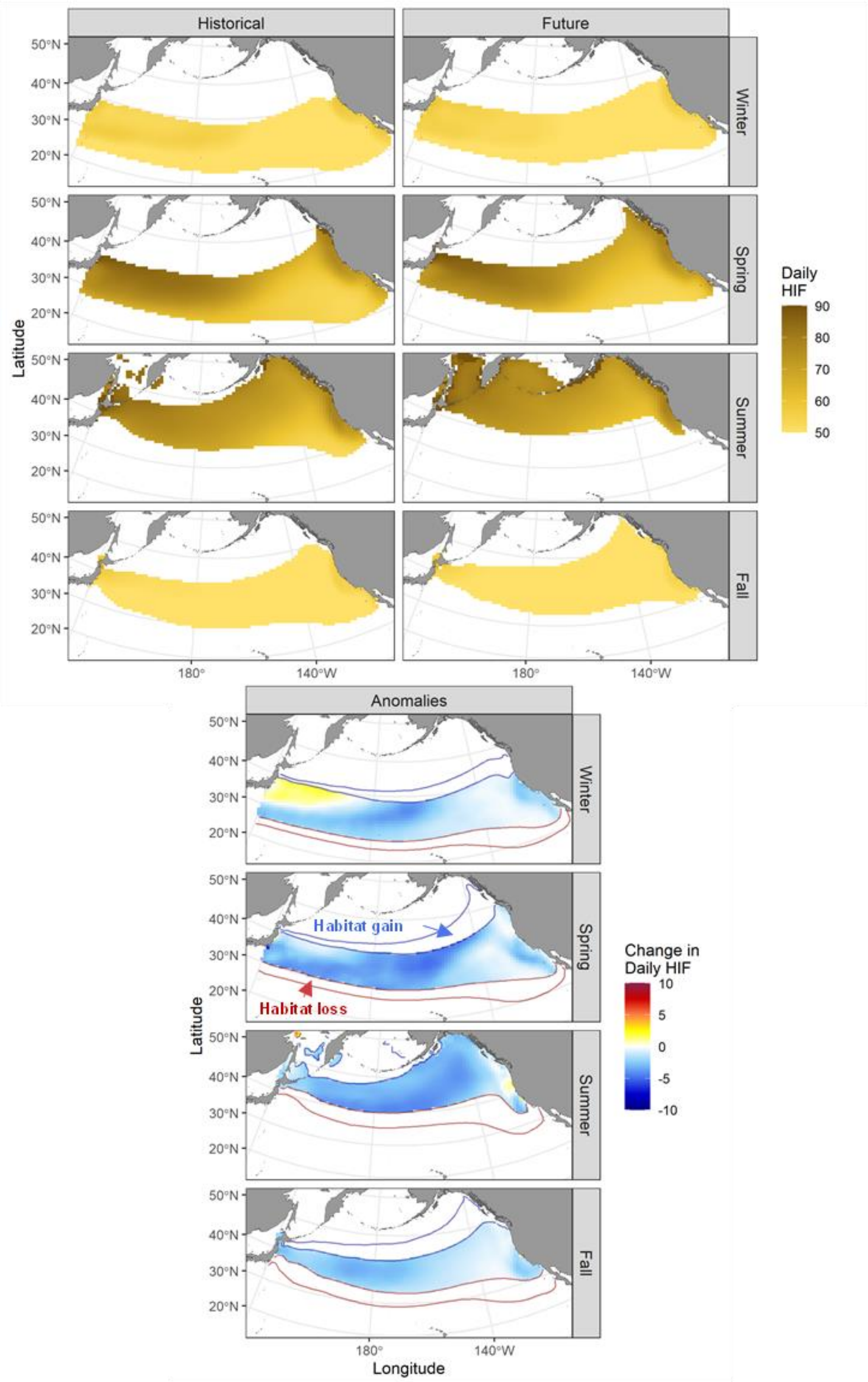

Supplementary Figure 11: Predictions of Heat Increment of Feeding (HIF) for the historical period (top left), future period (top right), and the predicted change between the two time periods (bottom). Areas where SST is outside albacore favorable thermal habitat (11 – 22 °C) are masked. The red lines show the historical and future locations of the 22 °C isotherm, thus areas outlined in red show where future warming results in loss of albacore favorable thermal habitat. The blue lines show the historical and

future locations of the 11 °C isotherm, thus areas outlined in blue show where future warming results in gain of albacore favorable thermal habitat.

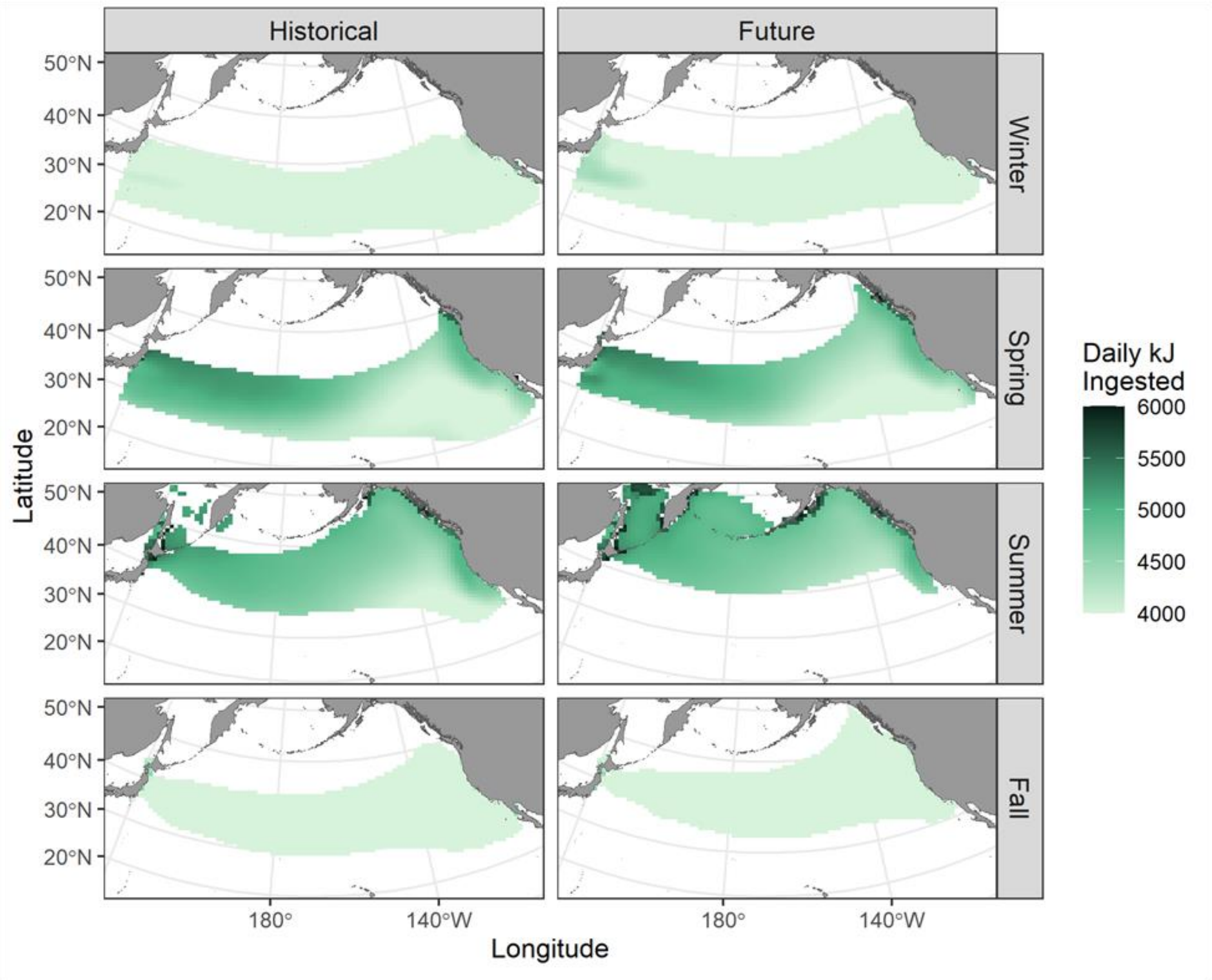

Supplementary Figure 12: Predictions of daily kJ ingested for the historical period (left) and future period (right). Areas where SST is outside albacore favorable thermal habitat (11 – 22 °C) are masked. Future anomalies are shown in the main text (Figure 6).

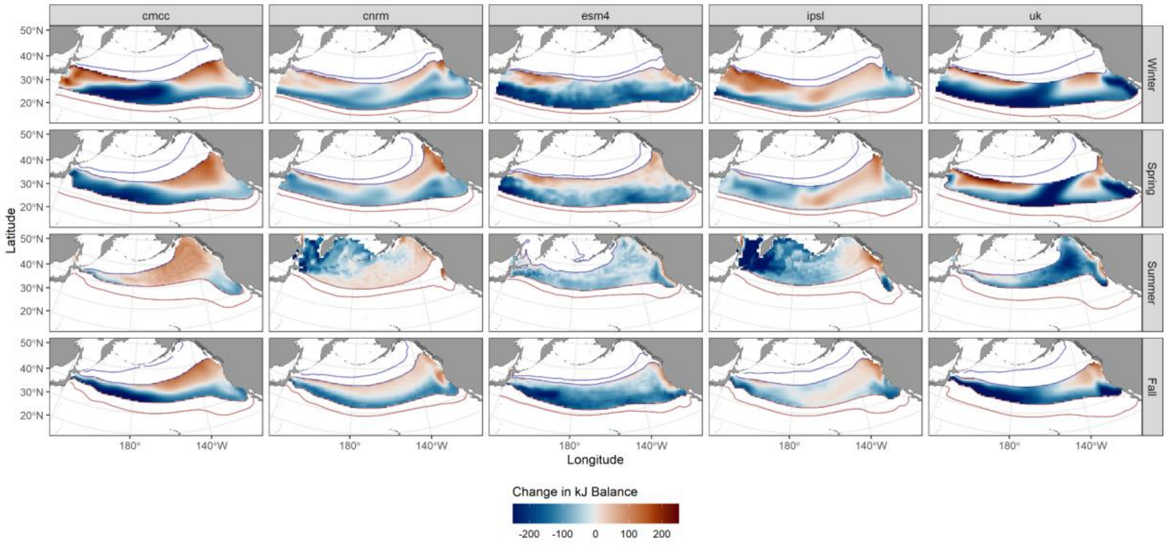

Supplementary Figure 13: Projected changes to kJ balance for each earth system model separately. Areas where SST is outside albacore favorable thermal habitat (11 – 22 °C) are masked. The red lines show the historical and future locations of the 22 °C isotherm, thus areas outlined in red show where future warming results in loss of albacore favorable thermal habitat. The blue lines show the historical and future locations of the 11 °C isotherm, thus areas outlined in blue show where future warming results in gain of albacore favorable thermal habitat. Changes in kJ balance are indicated as colored shading in the areas where habitat was thermally suitable in both the past and future time periods.

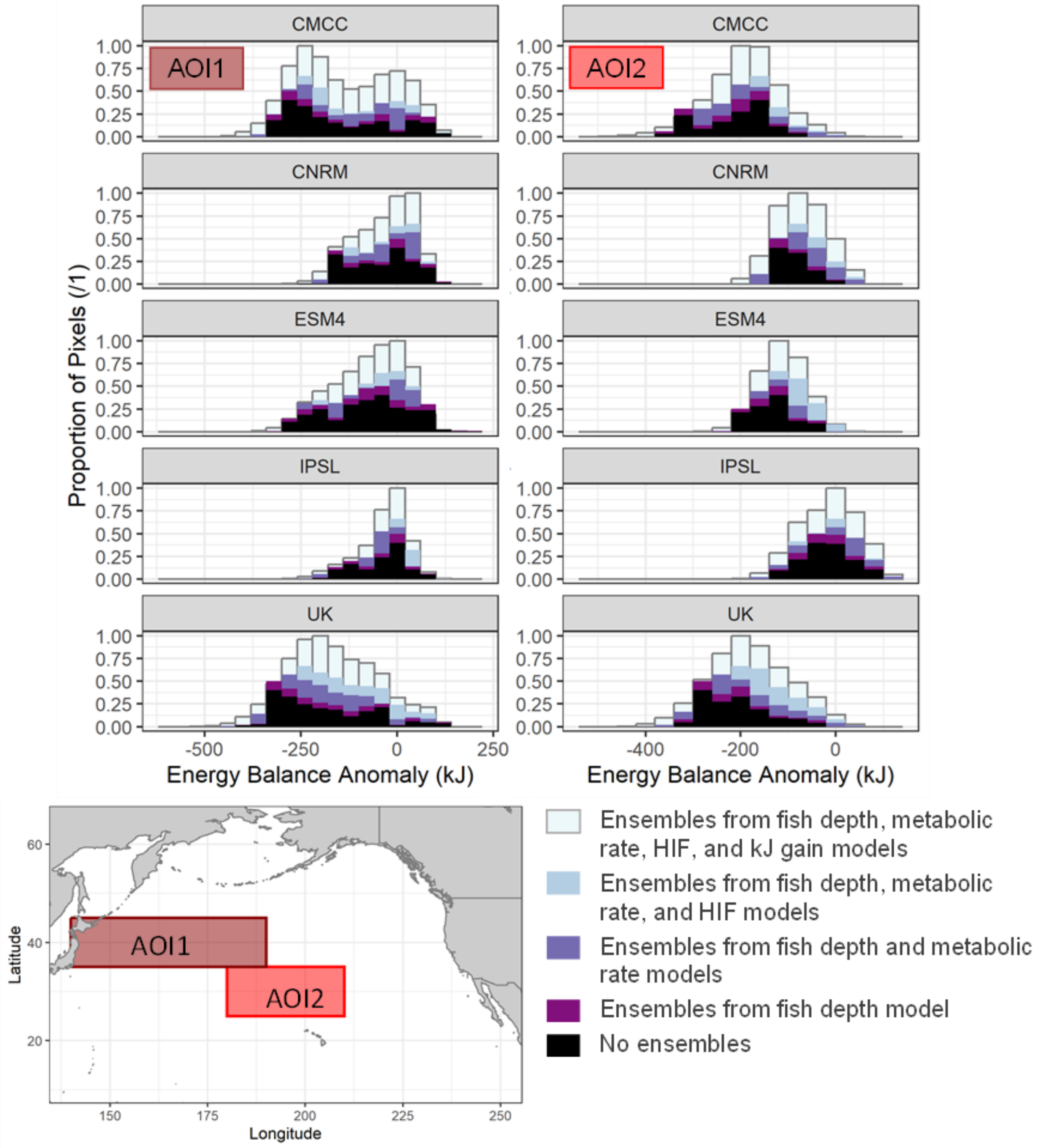

Supplementary Figure 14: Contribution of different models to overall uncertainty in projected kJ balance anomaly. Results are shown for two Areas of Interest (AOIs) with substantial future anomalies in kJ balance (see Figure 6). AOI1 shows anomalies in winter, AOI2 shows anomalies in fall. Uncertainty was quantified across each of the five earth system models (CMCC-ESM2, CNRM-ESM2-1, GFDL-ESM4, IPSL-CM6A-LR, UKESM1-0-LL: see Supplementary Table 1), and using an ensemble of 10 simulations each from the posterior distributions of each GAM. Each histogram shows the distribution of projected future anomalies within each AOI. The black bars show predictions without the use of GAM ensembles (i.e., equivalent results to those shown in Figure 6, with variability in kJ balance anomaly only from spatial variability within each AOI). Other colors show the sequential addition of ensembles for each of the four GAMs, with the lightest blue bars showing the range of predictions for the full model ensemble (10,000 models: 10 x 10 x 10 x 10). The height of the histogram bars is scaled so that predictions from each subset of the full ensemble are more visible.

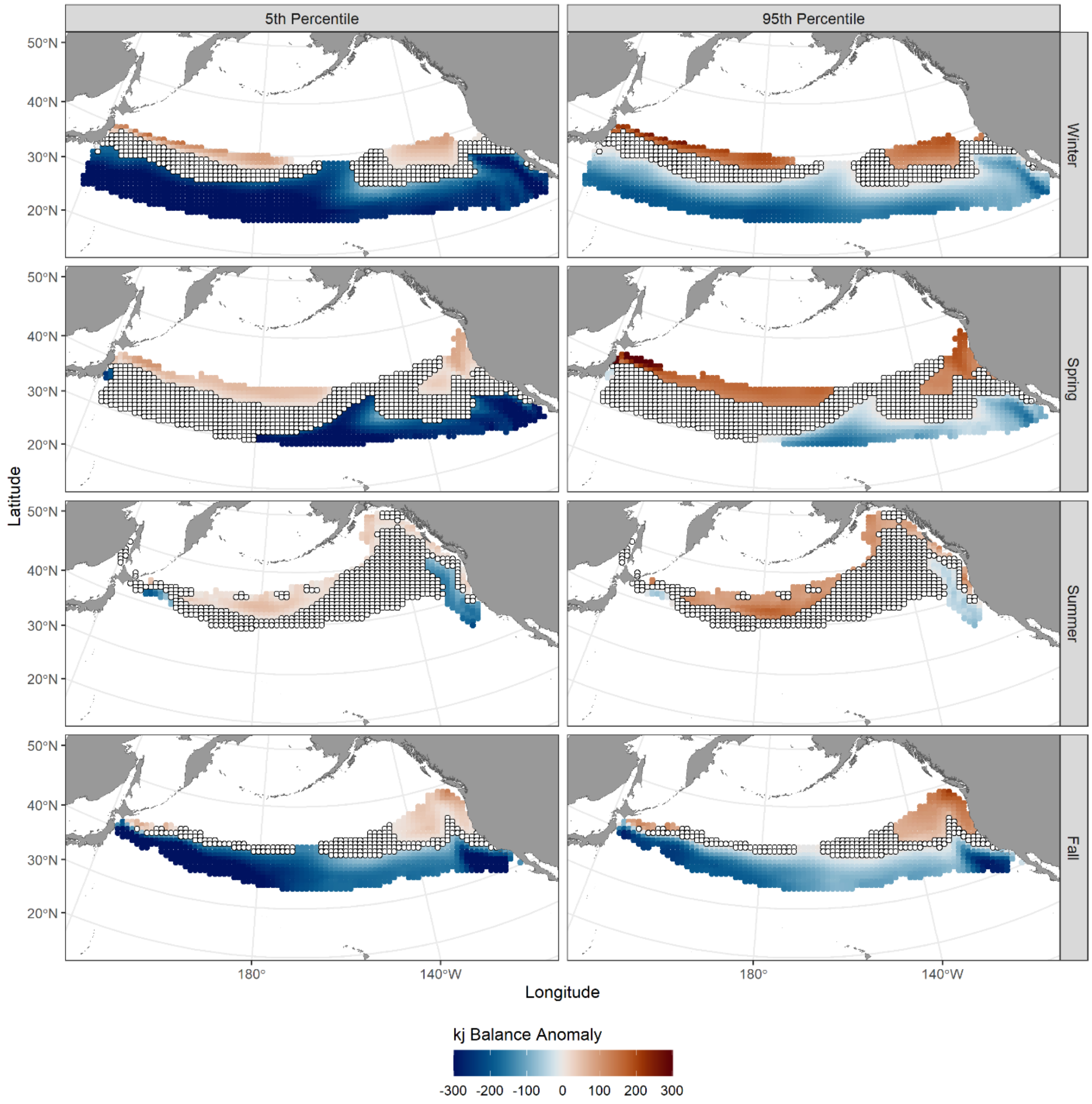

Supplementary Figure 15: Projected changes to kJ balance for the UKESM1-0-LL earth system model only. Shown are the 5th and 95th percentile of kJ balance anomalies from the ensemble of simulations depicted in Supplementary Figure 14. These percentiles thus capture the variability in projections resulting from the GAMs. White bordered cells represent those where the 5th and 95th percentile overlapped with 0 (or no change between the historical and future time-periods). Areas where SST is outside albacore favorable thermal habitat (11 – 22 °C) are masked. Changes in kJ balance are indicated as colored shading in the areas where habitat was thermally suitable in both the past and future time periods.

Supplementary References

Aumont, O., Ethé, C., Tagliabue, A., Bopp, L., & Gehlen, M. (2015). PISCES-v2: An ocean biogeochemical model for carbon and ecosystem studies. Geoscientific Model Development, 8, 2465–2513. https://doi.org/10.5194/gmd-8-2465-2015

Boucher, O,. Servonnat, J., Albright, A. L., Aumont, O., Balkanski, Y., Bastrikov, V., et al. 2020. Presentation and evaluation of the IPSL-CM6A-LR climate model. Journal of Advances in Modeling Earth Systems. https://doi.org/10.1029/2019MS002010

Brill, R. W. (1987). On the standard metabolic rates of tropical tunas, including the effect of body size and acute temperature change. Fishery Bulletin, 85(1), 25-35.

Dunne, J. P., Horowitz, L. W., Adcroft, A. J., Ginoux, P., Held, I. M., John, J. G., ... & Zhao, M. (2020). The GFDL Earth System Model version 4.1 (GFDL-ESM 4.1): Overall coupled model description and simulation characteristics. Journal of Advances in Modeling Earth Systems, 12(11), e2019MS002015.

Fujioka, K., Fukuda, H., Tei, Y., Okamoto, S., Kiyofuji, H., Furukawa, S., ... & Kitagawa, T. (2018). Spatial and temporal variability in the trans-Pacific migration of Pacific bluefin tuna (*Thunnus orientalis*) revealed by archival tags. Progress in Oceanography, 162, 52-65.

Galuardi, B., Royer, F., Golet, W., Logan, J., Neilson, J., & Lutcavage, M. (2010). Complex migration routes of Atlantic bluefin tuna (*Thunnus thynnus*) question current population structure paradigm. Canadian Journal of Fisheries and Aquatic Sciences, 67(6), 966-976.

Gleiber, M. R., Hardy, N. A., Roote, Z., Krug-MacLeod, A. M., Morganson, C. J., Tandy, Z., ... & Green, S. J. (2024). The Pelagic Species Trait Database, an open data resource to support trait-based ocean research. Scientific Data, 11(1), 2.

Graham, J. B., & Laurs, R. M. (1982). Metabolic rate of the albacore tuna *Thunnus alalunga*. Marine Biology, 72, 1-6.

Hijmans R (2022). _geosphere: Spherical Trigonometry_. R package version 1.5-18, <https://CRAN.R-project.org/package=geosphere>.

Lam, C. H., Nielsen, A., & Sibert, J. R. (2008). Improving light and temperature based geolocation by unscented Kalman filtering. Fisheries Research, 91(1), 15-25.

Lovato, T., Peano, D., Butenschön, M., Materia, S., Iovino, D., Scoccimarro, E., ... & Navarra, A. (2022). CMIP6 simulations with the CMCC Earth system model (CMCC-ESM2). Journal of Advances in Modeling Earth Systems, 14(3), e2021MS002814.

Nielsen, A., Sibert, J. R., Ancheta, J., Galuardi, B., and Lam, C. H. (2012). ukfsst: Kalman Filter tracking including Sea Surface Temperature. R package version 0.3.

Schulzweida, U. (2023). CDO User Guide. 2019. CDO User Guide (Version 1.9. 5). Zenodo. Available online: http://doi. org/10.5281/zenodo, 1435455.

Séférian, R., Nabat, P., Michou, M., Saint-Martin, D., Voldoire, A., Colin, J., et al. 2019. Evaluation of CNRM Earth-System model, CNRM-ESM2-1: role of Earth system processes in present-day and future

climate. Journal of Advances in Modeling Earth Systems, 11, 4182– 4227. https://doi.org/10.1029/2019MS001791

Sellar, A.A., Jones, C.G., Mulcahy, J., Tang, Y., Yool, A., Wiltshire, A., et al. 2019. UKESM1: Description and evaluation of the UK Earth System Model. Journal of Advances in Modeling Earth Systems, 11. https://doi.org/ 10.1029/2019MS0017392019

Stock, C. A., Alexander, M. A., Bond, N. A., Brander, K. M., Cheung, W. W., Curchitser, E. N., ... & Werner, F. E. (2011). On the use of IPCC-class models to assess the impact of climate on living marine resources. Progress in Oceanography, 88(1-4), 1-27.

Stock, C. A., Dunne, J. P., Fan, S., Ginoux, P., John, J., Krasting, J. P., ... & Zadeh, N. (2020). Ocean biogeochemistry in GFDL's Earth System Model 4.1 and its response to increasing atmospheric CO_2. Journal of Advances in Modeling Earth Systems, 12(10), e2019MS002043.

Vichi, M., Lovato, T., Butenschön, M., Tedesco, L., Lazzari, P., Cossarini, G., et al. (2020). The Biogeochemical Flux Model (BFM): Equation description and user manual. BFM version 5.2 (BFM Report series N. 1, Release 1.2, p. 104). Retrieved from http://bfm-community.eu

Yool, A., Popova, E. E., & Anderson, T. R. (2013). MEDUSA-2.0: An intermediate complexity biogeochemical model of the marine carbon cycle for climate change and ocean acidification studies. Geoscientific Model Development, 6(5), 1767–1811. https://doi.org/10.5194/gmd-6-1767-2013
